# Transcriptomic analysis reveals the gene expression profile that specifically responds to IBA during adventitious rooting in mung bean seedlings

**DOI:** 10.1186/s12864-016-2372-4

**Published:** 2016-01-12

**Authors:** Shi-Weng Li, Rui-Fang Shi, Yan Leng, Yuan Zhou

**Affiliations:** School of Environmental and Municipal Engineering, Key Laboratory of Extreme Environmental Microbial Resources and Engineering Gansu Province, Lanzhou Jiaotong University, 88 West Anning Road, Lanzhou, 730070 P. R. China; School of Chemical and Biological Engineering, Lanzhou Jiaotong University, 88 West Anning Road, Lanzhou, 730070 P.R. China

**Keywords:** *Vigna radiata* (L.) R.Wilczek, Adventitious root, Gene expression, Indole-3-butyric acid (IBA), Transcriptome, RNA-Seq

## Abstract

**Background:**

Auxin plays a critical role in inducing adventitious rooting in many plants. Indole-3-butyric acid (IBA) is the most widely employed auxin for adventitious rooting. However, the molecular mechanisms by which auxin regulate the process of adventitious rooting are less well known.

**Results:**

The RNA-Seq data analysis indicated that IBA treatment greatly increased the amount of clean reads and the amount of expressed unigenes by 24.29 % and 27.42 % and by 4.3 % and 5.04 % at two time points, respectively, and significantly increased the numbers of unigenes numbered with RPKM = 10-100 and RPKM = 500-1000 by 13.04 % and 3.12 % and by 24.66 % and 108.2 % at two time points, respectively. Gene Ontology (GO) enrichment analysis indicated that the enrichment of down-regulated GOs was 2.87-fold higher than that of up-regulated GOs at stage 1, suggesting that IBA significantly down-regulated gene expression at 6 h. The GO functional category indicated that IBA significantly up- or down-regulated processes associated with auxin signaling, ribosome assembly and protein synthesis, photosynthesis, oxidoreductase activity and extracellular region, secondary cell wall biogenesis, and the cell wall during the development process. Kyoto Encyclopedia of Genes and Genomes (KEGG) pathway enrichment indicated that ribosome biogenesis, plant hormone signal transduction, pentose and glucuronate interconversions, photosynthesis, phenylpropanoid biosynthesis, sesquiterpenoid and triterpenoid biosynthesis, ribosome, cutin, flavonoid biosynthesis, and phenylalanine metabolism were the pathways most highly regulated by IBA. A total of 6369 differentially expressed (2-fold change > 2) unigenes (DEGs) with 3693 (58 %) that were up-regulated and 2676 (42 %) down-regulated, 5433 unigenes with 2208 (40.6 %) that were up-regulated and 3225 (59.4 %) down-regulated, and 7664 unigenes with 3187 (41.6 %) that were up-regulated and 4477 (58.4 %) down-regulated were detected at stage 1, stage 2, and between stage 1 and stage 2, respectively, suggesting that IBA treatment increased the number of DEGs. A total of 143 DEGs specifically involved in plant hormone signaling and 345 transcription factor (TF) genes were also regulated by IBA. qRT-PCR validation of the 36 genes with known functions indicated a strong correlation with the RNA-Seq data.

**Conclusions:**

The changes in GO functional categories, KEGG pathways, and global DEG profiling during adventitious rooting induced by IBA were analyzed. These results provide valuable information about the molecular traits of IBA regulation of adventitious rooting.

**Electronic supplementary material:**

The online version of this article (doi:10.1186/s12864-016-2372-4) contains supplementary material, which is available to authorized users.

## Background

Adventitious roots refer to roots that form from any tissue that is not a root, such as leaves and stems. This is part of the normal development of a plant and occurs naturally or can also be induced by stresses such as wounding, flooding, and etiolation [1]. Under environmental stress, adventitious rooting acts as an alternative or supplement to seed propagation and is an important mechanism in response to stresses [2]. This method of vegetative propagation in plants has been widely used for the commercial production of woody forest and horticultural species [3]. The formation of adventitious roots has been associated with important aspects of tissue dedifferentiation and can be induced reproducibly under the control of exogenous phytohormones. Auxin acts as an effective inducer of adventitious roots in many plants and may interact with other endogenous factors or environmental stimuli during this process [4–6]. Horticultural and agricultural practices have demonstrated that the external application of auxin exerts a great effect in inducing adventitious rooting in many plant species. Indole-3-butyric acid (IBA) is used worldwide as a rooting hormone due to its higher stability and efficiency versus indole 3-acetic acid (IAA). In cells, IBA converts into IAA and acts as a slow release source of IAA [1, 7, 8]. Auxin can induce ethylene production via the up-regulation of the 1-aminocyclopropane-1-carboxylic acid (ACC) synthase 4 (*ACS4*) gene, which was found to be an early auxin-induced gene [9, 10], to further promote adventitious rooting [11]. Although auxin plays a crucial role in inducing cell dedifferentiation and root primordium formation, the exact mechanism of its action is still poorly understood.

The physiological and biochemical changes that occur during the complex process of *in vitro* root development have been extensively investigated in last few decades. However, the molecular mechanisms underlying this process must be further explored. In recent years, various molecular and genetic approaches have been used to identify the genes involved in regulating adventitious root development in *Arabidopsis* and other plants. Among the genes identified, several gene families in the auxin signaling pathway have been shown to mediate adventitious rooting in many plants, such as the members of the auxin-inducible *ARF* (*AUXIN RESPONSE FACTOR*) family [12], members of the auxin-inducible *GH3* (*GRETCHEN HAGEN 3*) family [13], the tryptophan-dependent IAA biosynthesis gene *YUCCA* [14], the auxin efflux carrier genes *PIN* (*PIN-FORMED*) and *ABCB/PGP* (*ATP BINDING CASSETTE-TYPE B/ P-GLYCOPROTEINs*) [15–17], the auxin influx carrier gene *AUX1/LAX* (*AUXIN/IAA*) [15, 16], and the auxin-responsive LOB-domain (*LATERAL ORGAN BOUNDARIES-DOMAIN*) genes [18]. LOB-domain transcription factors encoded by several phylogenetically, closely related genes, such as the rice genes *ARL1* (*ADVENTITIOUS ROOTLESS1*) [19] and *CRL1* (*CROWN ROOTLESS1*) [20], the maize gene *RTCS* (*ROOTLESS CONCERNING CROWN AND SEMINAL ROOTS*) [21], the *Medicago* gene *MtLOB29* [22], and the *Arabidopsis* genes *LBD16* (*LATERAL ORGAN BOUNDARIES-DOMAIN*) and *LBD29* [23], have been identified and shown to mediate adventitious rooting. Moreover, the *SCARECROW*-like genes [24], the cytokinin type B response regulator (*PtRR13*) gene [25], and the *APETALA2/ETHYLENE RESPONSE FACTOR* (*AP2/ERF*) transcription factor genes [26] have also been demonstrated to be involved in modulating adventitious rooting. Using cDNA microarrays, Brinker et al. (2004) identified 220 genes that were significantly changed during root development in hypocotyl cuttings of *Pinus contorta* under IBA treatment [27]. Holmes et al. (2010) identified 904 and 993 up- and down-regulated probe sets in root-forming cultures of *Medicago truncatula* as well as significant changes in metabolism, signaling and the expression of transcription factors linked to *in vitro* adventitious root formation processes [22]. Despite this, the gene expression profile during adventitious rooting in response to auxin remains uncharacterized.

Recently, RNA-Seq has been used to explore transcriptomic data and study gene expression at the whole genome level in model and non-model organisms [28]. *De novo* short read assembly technology has been successfully applied to identify gene expression profiles and discover new genes without a reference genome sequence [29]. This technology platform enables the precise elucidation of transcripts present within a particular sample and can be used to calculate gene expression based on absolute transcript abundance [30]. This technology has also been used to investigate the gene expression profile during adventitious rooting. Wei et al. (2014) reported 1091 differentially expressed unigenes, including 656 up- and 435 down-regulated genes, in tea cuttings (*Camellia sinensis*) treated with IBA. The genes involved in plant hormone signal transduction, secondary metabolism, cell wall organization, and glutathione metabolism potentially contribute to adventitious rooting [31].

Mung bean is one of the most important tropical grain legumes that serves as a significant and a cheap source of carbohydrates and easily digestible protein for the people of Asia and Africa but is also increasingly extending into Australia, USA, Canada and Ethiopia [32]. Furthermore, this plant has been widely used as a model plant species to study physiological, biochemical, and molecular mechanisms involved in the process of adventitious root formation [4, 9, 11, 33–36]. This provides an ideal experimental system with which to study the entire process of root formation, including the pre-morphogenesis stages [37]. In our previous studies, we had demonstrated that IBA has a great effect on promoting adventitious rooting in mung bean seedlings [38]. Recently, we had characterized the mung bean transcriptome and the differentially expressed gene profile and metabolic pathways during early stage (the induction and initiation stages) of adventitious root development in the hypocotyl cuttings without auxin treatment using RNA-Seq technology [39]. In the present study, RNA-Seq technology was exploited to highlight global changes in gene expression in response to IBA during the induction (6 h) and initiation (24 h) stages of root development in mung bean hypocotyl cuttings. We aimed to perform gene profiling, characterize the metabolic pathways specifically regulated by IBA and further to uncover the molecular basis of IBA promoting adventitious rooting. Real-time quantitative PCR was used to validate several of the transcriptional changes observed.

## Results and discussion

### Illumina Solexa RNA paired-end sequencing and mapped reads

**Fig. 1:**
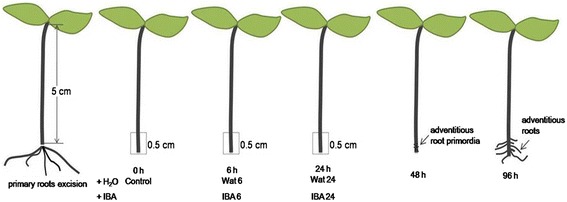
Time course of adventitious root development in mung bean hypocotyls after the primary root excision. Adventitious root primordia are visible at 48 h after primary root excision, and adventitious roots grow through the epidermis of the hypocotyls within 96 h. The basal 0.5 cm of the hypocotyls at 0 h, 6 h (stage 1), and 24 h (stage 2) after primary root excision and treatment with water or 10 μM IBA were harvested and used as study samples

To provide a comprehensive overview of mung bean and adventitious rooting in the seedlings at a transcriptional level, five cDNA libraries were constructed and sequenced from the hypocotyl tissues of 5 day-old seedlings harvested separately at 0 h (Con), after primary root excision and incubation in water for 6 h (Wat6) or 10 μM IBA for 6 h (IBA6), and after primary root excision and incubation in water for 24 h (Wat24) or IBA for 24 h (IBA24) (Fig. 1), of which, the Con, Wat6, and Wat24 were previously sequenced in our previous study [39]. The sequencing data and mapping results of the cDNA libraries of IBA6 and IBA24 were listed in Additional file 1 and Table 1. On average, 94 % of the quality filter passed reads generated for all five samples was mapped uniquely to the reference sequences. Data analysis showed that the amounts of raw reads and clean reads were increased by 24.28 % and 24.29 % at stage 1, respectively, and increased by 23.93 % and 27.42 % at stage 2, respectively, reflecting a great increase in the number of sequences resulting from IBA treatment.

**Table 1:** Sample mapping results and unigene abundance measurements in the samples

	IBA6	IBA24
Clean reads	69082097	70805846
Mapped reads	64711415	66557328
Mapped ratio	93.67 %	94.00 %
All unigenes	78697	78697
Expressed unigenes	69527	67938
Expressed ratio	88.35 %	86.33 %
RPKM ≥ 1000 unigenes	41	46
RPKM 500-1000 unigenes	91	127
RPKM 100-500 unigenes	957	976
RPKM 10-100 unigenes	9824	9613
RPKM 1-10 unigenes	15535	14053
RPKM <1 unigenes	43076	43120
Maxim RPKM	24455	9746
Average RPKM	10.08	10.44

### *De novo* assembly, unigene determination and abundance measurement

The paired-end *de novo* assembly of the processed reads was performed using the TRINITY transcriptome assembly software program, which can recover more full-length transcripts across a broad range of expression levels and provides a unified, sensitive solution for transcriptome reconstruction in species without a reference genome, similar to methods that rely on genome alignments [40]. The Chrysalis cluster module of TRINITY was used to cluster the transcripts and generated a total of 78,697 unigenes [39]. The ratios of expressed unigenes were 84.71 % (66,663), 88.35 % (69,527), 82.19 % (64,680), and 86.33 % (67,938) in Wat6, IBA6, Wat24, and IBA24, respectively. These results indicate that the amounts of unigenes expressed at stage 1 and stage 2 were increased by 4.3 % (2864 unigenes) and 5.04 % (3258 unigenes), respectively. Clearly, IBA enhanced the number of genes expressed during the early stages of rooting (Table 1).

To evaluate the abundances of the expressed unigenes, gene expression levels were estimated from Illumina sequencing based on the number of clean reads for a gene. The RPKM method [30] was used to calculate the expression abundances of unigenes in each sample. The results showed that the unigenes numbered with RPKM = 10-100 were significantly increased by 13.04 % at stage 1 and by 3.12 % at stage 2. The unigenes numbered with RPKM = 500-1000 were significantly increased by 24.66 % at stage 1 and by 108.2 % at stage 2. These results suggest that the expression abundances of certain genes were greatly increased by IBA treatment (Table 1).

### GO enrichment analysis

**Fig. 2:**
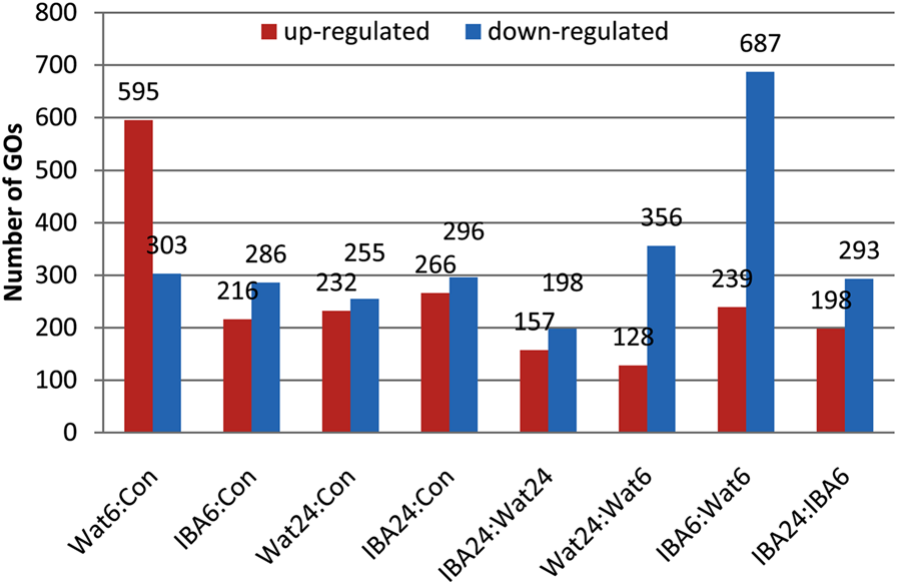
The distribution of GO terms enriched in the sample pairs

To discern global patterns of differential transcript abundance over the time course, unigenes with contrasting significance at FDR < 0.05 were further filtered to include only those with greater than 2-fold changes (log2 > 1) in unigene abundance between the samples. Unigenes meeting these criteria were clustered using Blast2GO software (version 2.3.5, http://www.blast2go.de/) [41] and WEGO software [42]. To evaluate the GOs differentially regulated during the time course, the significantly up- and down-regulated GOs were enriched between IBA6 and Con and between IBA24 and Con. The results showed that the up- and down-regulated enriched GOs decreased by 63.7 % (379 GOs) and 5.6 % (17 GOs) at stage 1, respectively, and increased by 14.7 % (34 GOs) and 16.1 % (41 GOs) at stage 2, respectively. Furthermore, the up- and down-regulated enriched GOs decreased by 34.3 % (82 GOs) and 71.2 % (489 GOs), respectively, from stage 1 to stage 2. The down-regulated enriched GOs were 2.87-fold (448 GOs) higher than that of the up-regulated at stage 1, while the down-regulated enriched GOs were increased by 26.1 % (41 GOs) compared to the up-regulated GOs at stage 2 (Fig. 2). These results indicate that the majority of GOs were significantly down-regulated at stage 1, suggesting IBA treatment significantly down-regulated gene expression after 6 h of adventitious rooting. The results further suggest that profound cellular and metabolic reorganization occurs with 6 h of rooting.

**Fig. 3:**
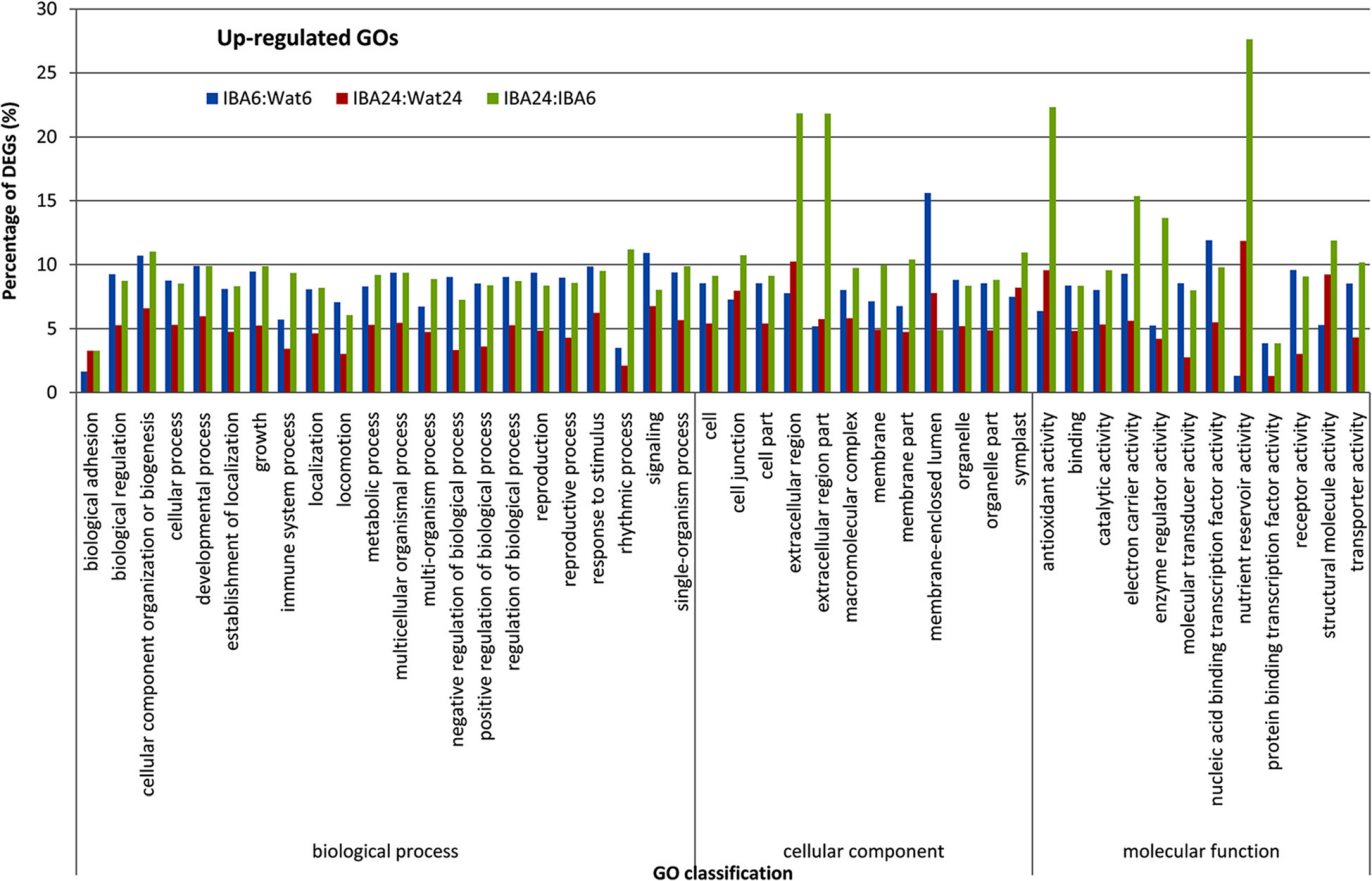
GO enrichment for up-regulated unigenes

**Fig. 4:**
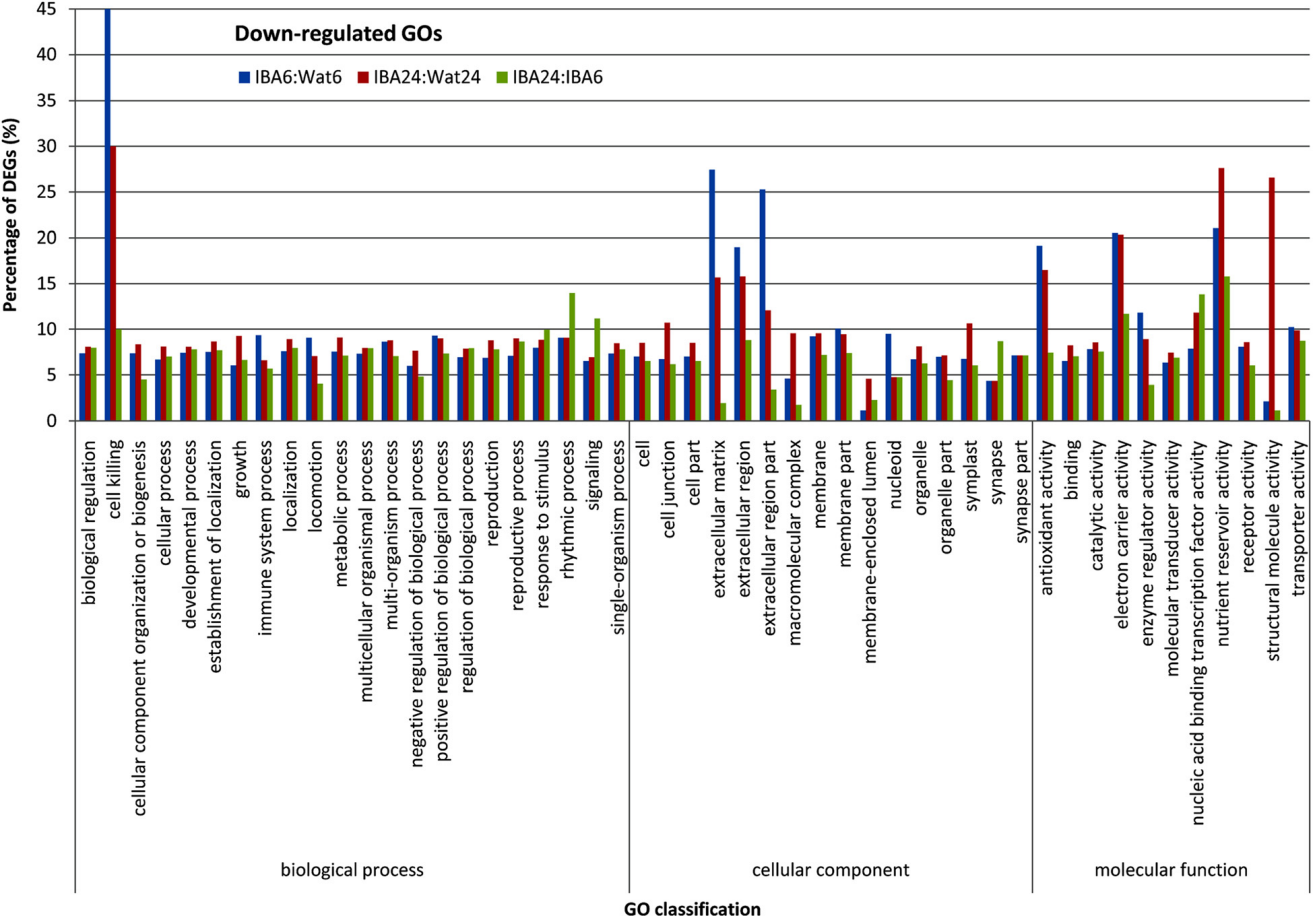
GO enrichment for down-regulated unigenes

The differentially regulated GO subcategories are presented in Figs. 3 and 4. In the group of up-regulated GO terms, the percentages of differentially expressed genes (DEGs) in most of the GO subcategories at stage 1 were higher than those at stage 2 with the exceptions of nutrient reservoir activity and structural molecule activity in molecular function, suggesting that most of the DEGs were significantly up-regulated at 6 h after IBA treatment. Furthermore, the percentage of DEGs clustered into the categories of rhythmic process in biological process, extracellular region and extracellular region part in cellular component, and antioxidant activity, electron carrier activity, enzyme regulator activity, and nutrient reservoir activity in molecular function were strongly increased from stage 1 to stage 2. In the group of down-regulated GO terms, nearly equal percentages of DEGs in most of the subcategories were examined between stage 1 and stage 2, with the exception of cell killing in biological process, extracellular matrix, extracellular region, and extracellular region part in cellular component, nutrient reservoir activity, and structural molecule activity in molecular function. In the subcategories of cell killing, extracellular matrix, extracellular region, and extracellular region part, the DEGs ratio at stage 1 was higher than that at stage 2, whereas for nutrient reservoir activity and structural molecule activity, the DEGs ratio at stage 1 was lower than that at stage 2. These results suggest that profound changes in biological function caused by IBA occurred in GO subcategories such as extracellular region, extracellular matrix, extracellular region part, antioxidant activity, electron carrier activity, cell killing, and structural molecule activity during early stages of IBA-induced rooting.

Additional file 2 and Table 2 list the top 50 significantly up- and down-regulated GO categories during the IBA-induced development process. The results revealed that the GOs associated with auxin signaling, such as auxin mediated signaling pathway, cellular response to auxin stimulus, hormone-mediated signaling pathway, response to auxin stimulus, cellular response to hormone stimulus, and hormone-mediated signaling pathway, were significantly up-regulated both at stage 1 and stage 2. The GOs associated with ribosome assembly and protein synthesis, such as nucleolus, ribonucleoprotein complex biogenesis, ribosome biogenesis, rRNA metabolic process, rRNA processing, ribosome, structural constituent of ribosome, ribosomal subunit, cytosolic ribosome, and translation, were significantly up-regulated at stage 1 but significantly down-regulated at stage 2. The GOs associated with photosynthesis, such as photosystem, photosynthesis, photosystem II, and photosystem I, were significantly down-regulated at stage 1. The GOs for oxidoreductase activity and extracellular region were significantly down-regulated both at stage 1 and stage 2. The GO secondary cell wall biogenesis was down-regulated at stage 1, while the GO cell wall was up-regulated at stage 2. The GOs associated with photosynthesis, such as light reaction, photosystem, photosystem II, and thylakoid, as well as cell wall, external encapsulating structure, and hydrolase activity (hydrolyzing O-glycosyl compounds), were significantly up-regulated from stage 1 to stage 2. However, the GOs associated with hormone pathway and signaling such as response to chemical stimulus, response to hormone stimulus, hormone-mediated signaling pathway, response to organic substance, cellular response to hormone stimulus, response to auxin stimulus, cellular response to endogenous stimulus, nucleic acid binding transcription factor activity, and sequence-specific DNA binding transcription factor activity, were significantly down-regulated from stage 1 to stage 2.

**Table 2:** Top-5 significantly up- and down-regulated GOs between the samples

GO ID	Total genes	DEGs	FDR	Level	Terms
IBA6:Wat6					
Down-regulated					
GO:0005576	1222	232	5.87E-38	2	extracellular region
GO:0016491	1856	291	1.34E-32	3	oxidoreductase activity
GO:0009521	79	49	3.74E-32	3	photosystem
GO:0015979	192	72	3.66E-29	4	photosynthesis
GO:0046906	485	119	7.40E-29	4	tetrapyrrole binding
Up-regulated					
GO:0005730	378	103	1.06E-24	5	nucleolus
GO:0022613	200	70	2.29E-23	4	ribonucleoprotein complex biogenesis
GO:0042254	186	67	2.73E-23	5	ribosome biogenesis
GO:0009734	238	72	4.81E-20	6	auxin mediated signaling pathway
GO:0071365	247	72	4.35E-19	6	cellular response to auxin stimulus
IBA24:Wat24					
Down-regulated					
GO:0005840	538	161	2.51E-42	4	ribosome
GO:0003735	471	147	4.33E-41	3	structural constituent of ribosome
GO:0005198	606	161	1.42E-35	2	structural molecule activity
GO:0006412	671	157	8.65E-28	6	translation
GO:0030529	801	165	9.85E-23	3	ribonucleoprotein complex
Up-regulated					
GO:0009734	238	54	6.20E-17	6	auxin mediated signaling pathway
GO:0071365	247	55	6.20E-17	6	cellular response to auxin stimulus
GO:0009733	367	68	7.03E-17	5	response to auxin stimulus
GO:0005618	654	87	1.06E-12	4	cell wall
GO:0030312	671	87	3.91E-12	3	external encapsulating structure
IBA24:IBA6					
Down-regualted					
GO:0042221	2287	297	6.01E-21	3	response to chemical stimulus
GO:0009719	1364	204	1.28E-20	3	response to endogenous stimulus
GO:0009725	1235	182	1.63E-17	4	response to hormone stimulus
GO:0009755	768	130	5.55E-17	5	hormone-mediated signaling pathway
GO:0010033	1584	215	5.55E-17	4	response to organic substance
Up-regulated					
GO:0005576	1222	267	5.64E-39	2	extracellular region
GO:0015979	192	65	6.90E-18	4	photosynthesis
GO:0005618	654	133	2.56E-15	4	cell wall
GO:0030312	671	134	7.04E-15	3	external encapsulating structure
GO:0019684	82	34	7.93E-12	5	photosynthesis, light reaction

### KEGG pathway enrichment analysis

To further determine which biological pathways were significantly (FDR ≤ 0.05) modulated during the process, KEGG pathway enrichment was performed using the KEGG Automatic Annotation Server (KAAS) [43]. This analysis revealed that 12 and five pathways were enriched at stage 1 and stage 2, respectively. Among these, five KOs were significantly down-regulated and two KOs were significantly up-regulated at stage 1, suggesting that significant metabolic changes occur at the 6-h time point (Table 3, Additional file 3). Further analysis indicated that the pathways of ribosome biogenesis in eukaryotes and plant hormone signal transduction were up-regulated both at stage 1 and stage 2, and the pathway of pentose and glucuronate interconversions, which has a function in glucuronate and pectin degradation, was up-regulated at stage 2. The significantly down-regulated KOs at stage 1 included photosynthesis, photosynthesis-antenna proteins, phenylpropanoid biosynthesis, and sesquiterpenoid and triterpenoid biosynthesis, while those at stage 2 were ribosome, phenylpropanoid biosynthesis, cutin, flavonoid biosynthesis, and phenylalanine metabolism. When compared with stage 1, photosynthesis, phenylpropanoid biosynthesis, starch and sucrose metabolism, cell cycle, and ribosome were up-regulated, while plant hormone signal transduction, diterpenoid biosynthesis, aminobenzoate degradation, bisphenol degradation, glucosinolate biosynthesis, and phenylalanine metabolism were down-regulated from stage 1 to stage 2. The phenylpropanoid biosynthesis pathway is required for suberin and lignin biosynthesis. The cutin pathway is involved in suberin and wax biosynthesis. Flavonoids are a major class of plant secondary metabolites that serve multitude of functions, including as pigments and in antioxidant activity. The pathways of aminobenzoate degradation and bisphenol degradation have functions in xenobiotics biodegradation and metabolism. These results indicate that the plant hormone signal transduction and ribosome biogenesis pathways were significantly up-regulated by IBA treatment, photosynthesis activity was down-regulated at stage 1 but up-regulated at stage 2, and plant hormone signal transduction was down-regulated from stage 1 to stage 2. Furthermore, the pectin degradation pathway was up-regulated and suberin, lignin and wax biosynthesis were down-regulated at both stage 1 and stage 2, suggesting the beginning of cell wall loosening from stage 1 to stage 2. The secondary metabolism pathways, such as phenylpropanoids and associated flavonoids, might play important roles in IBA-induced adventitious rooting. Phenylpropanoids function in plant defense as inducible chemical barriers or as signaling molecules. The core phenylpropanoid pathway is organized from phenylalanine to an activated (hydroxy) cinnamic acid derivative, and the specific branch pathways lead to the formation of lignin, sinapate esters, condensed tannins, anthocyanins, coumarins, benzoic acids and flavonoids/isoflavonoids [44–46]. The common derivatives from the phenylpropanoid pathway, specifically phenolic acids, flavonoids, and lignin [47], are crucial regulators in cell division and differentiation [48] and stimulate in vitro rooting [49].

**Table 3:** Top most significantly up- and down-regulated KOs between the samples

KO ID	Total genes	DEGs	FDR ≤ 0.05	Description
IBA6:Wat6				
Down-regulated				
ko00195	53	31	1.29E-20	Photosynthesis
ko00196	15	12	5.01E-10	Photosynthesis- antenna proteins
ko00940	69	18	5.25E-05	Phenylpropanoid biosynthesis
ko00909	7	5	0.0013	Sesquiterpenoid and triterpenoid biosynthesis
Up-regulated				
ko03008	73	32	1.20E-14	Ribosome biogenesis in eukaryotes
ko04075	130	20	0.0256	Plant hormone signal transduction
IBA24:Wat24				
Down-regulaed				
ko03010	337	115	1.37E-41	Ribosome
ko00940	69	18	0.0016	Phenylpropanoid biosynthesis
ko00073	12	6	0.0176	Cutin
ko00941	17	7	0.0176	Flavonoid biosynthesis
ko00360	57	13	0.0408	Phenylalanine metabolism
Up-regulated				
ko03008	73	17	5.09E-06	Ribosome biogenesis in eukaryotes
ko03010	337	38	2.91E-05	Ribosome
ko04075	130	18	0.0020	Plant hormone signal transduction
ko00040	49	9	0.0164	Pentose and glucuronate interconversions
IBA24:IBA6				
Down-regulated				
ko04075	130	24	0.0020	Plant hormone signal transduction
ko00904	8	5	0.0083	Diterpenoid biosynthesis
ko00627	29	9	0.0084	Aminobenzoate degradation
ko00363	20	7	0.0154	Bisphenol degradation
ko00966	6	4	0.0154	Glucosinolate biosynthesis
Up-regulated				
ko00195	53	19	5.30E-06	Photosynthesis
ko00940	69	17	0.0033	Phenylpropanoid biosynthesis
ko00500	119	23	0.0067	Starch and sucrose metabolism
ko04110	94	19	0.0117	Cell cycle
ko03010	337	47	0.0142	Ribosome

### Global changes in differentially expressed genes (DEGs) during adventitious rooting in response to IBA

**Fig. 5:**
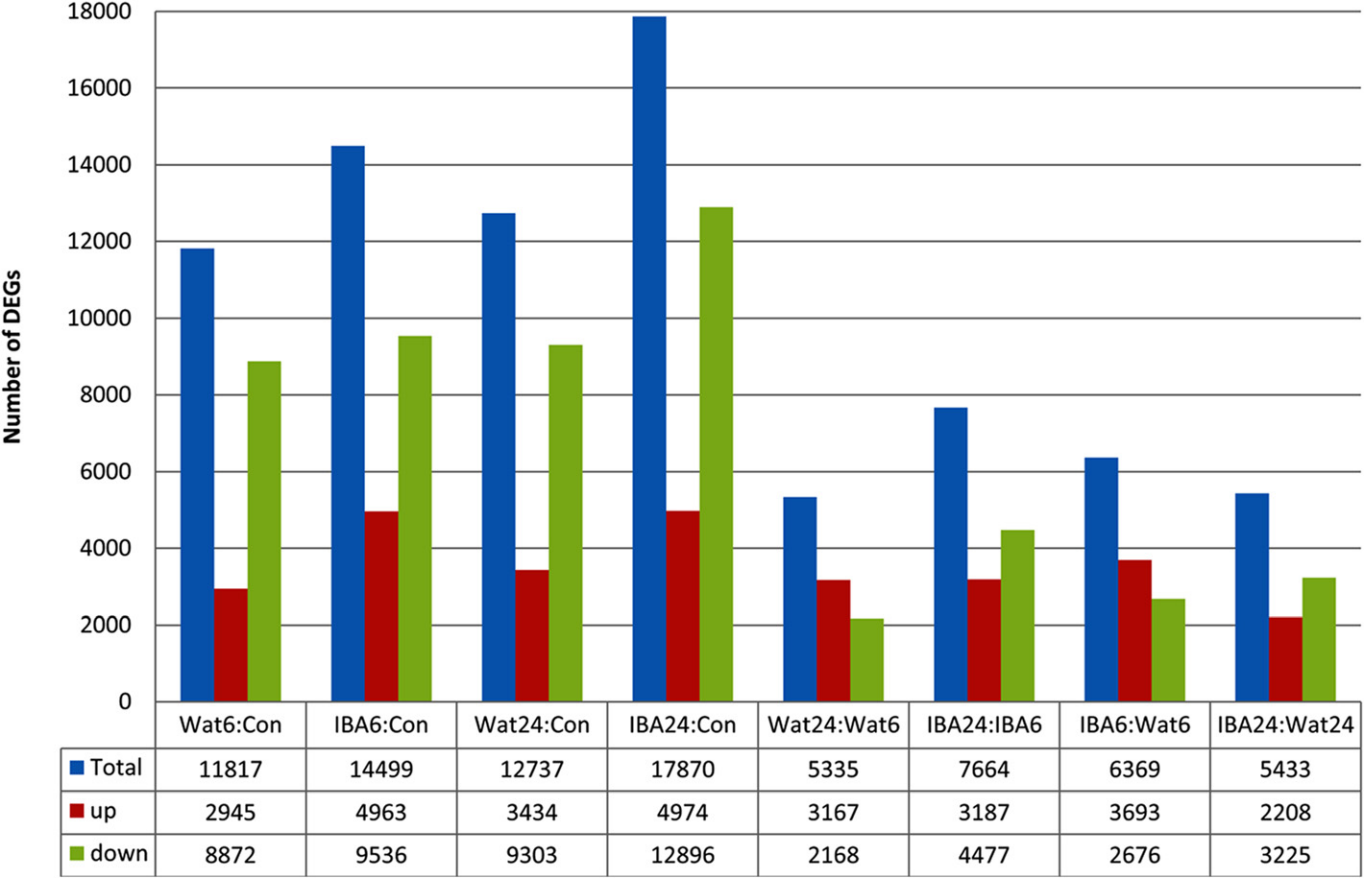
The distribution of DEGs in the sample pairs

To examine a gene expression profile during rooting under IBA treatment, the clean reads for a gene were mapped back to the assembled unigenes using BWA-0.6.2 software. The number of reads mapped to each unigene was then counted and normalized using RPKM [50]. Gene expression values were measured using the method described by DEGseq R package [51]. Of these, 6369 unigenes specifically with 3693 (58 %) up-regulated and 2676 (42 %) down-regulated, 5433 unigenes with 2208 (40.6 %) up-regulated and 3225 (59.4 %) down-regulated, and 7664 unigenes with 3187 (41.6 %) up-regulated and 4477 (58.4 %) down-regulated were differentially expressed (log2 ≥ 1) at stage 1, stage 2, and between stage 1 and stage 2, respectively. When compared with the control (Con), the number of DEGs (log2 ≥ 1) exhibited the following trend: IBA24 > IBA6 > Wat24 > Wat6. The DEGs number was increased by 22.7 % at stage 1 and by 40.3 % at stage 2. Clearly, IBA treatment increased the number of DEGs. Moreover, the down-regulated DEGs accounted for 75.1 %, 65.8 %, 73.0 %, and 72.2 % in Wat6, IBA6, Wat24, and IBA24, respectively, indicating the dominance of significantly down-regulated DEGs during the adventitious rooting process. The up-regulated DEGs accounted for 58.0 % and 40.6 % at stage 1 and stage 2, respectively, and total DEGs was increased by 17.2 % in IBA6 compared with IBA24. This result indicates that IBA treatment significantly up-regulated gene expression, and the greatest changes in gene expression occurred at the 6-h time point (Fig. 5 and Table 4). Further analysis revealed that the DEGs with 2 > log2 ≥ 1 accounted for 62.8 % and 60.8 % at stage 1 and stage 2, respectively, and the DEGs with log2 ≥ 5 accounted for 1.4 % and 1.5 % at stage 1 and stage 2, respectively. This indicates that the DEGs with 2- to 4-fold changes accounted for more than 60 % of the total, and there was no correlation between DEGs number and the time of IBA treatment (Table 4).

**Table 4:** Statistics of the DEGs between the time points during adventitious rooting

Samples		Log2							Total DEGs
		Total	NA	≥5	≥4	≥3	≥2	≥1	
IBA6:Wat6	down	2676	177	60	116	249	551	1523	6369
	up	3693	169	32	95	265	653	2479	
IBA24:Wat24	down	3225	239	56	132	345	627	1826	5433
	up	2208	139	24	64	163	340	1478	
IBA24:IBA6	down	4477	356	73	173	447	1051	2377	7664
	up	3187	293	65	116	308	698	1707	

### Gene expression patterns in response to IBA during the early stages of adventitious rooting

To gain insight into the gene expression patterns that occur in response to IBA during adventitious rooting, transcript levels of annotated unigenes were compared between different developmental time-points as well as between treatments. After filtering out the unigenes designated as unknown, hypothetical protein, uncharacterized protein, predicted protein, and unnamed protein in the database, the most significantly and abundantly expressed DEGs with both RPKM > 10 and log2 > 3 were selected and are listed in Additional files 4, 5 and 6.

Among the most significantly and abundantly expressed unigenes at stage 1, 17 DEGs with RPKM >10 were up-regulated 16- to 82-fold and 41 DEGs with RPKM >10 were up-regulated 8- to 16-fold. Thirty seven DEGs with RPKM >10 were down-regulated 16- to 222-fold and 23 unigenes with RPKM >10 were down-regulated 8- to 16-fold. Of those at stage 2, 13 DEGs with RPKM > 10 were up-regulated 16- to 63-fold and 23 DEGs with RPKM > 10 were up-regulated 8- to 16-fold. Twenty three DEGs with RPKM > 10 were down-regulated 16- to 388-fold, and 31 DEGs with RPKM > 10 were down-regulated 8- to 16- fold. The changes in DEGs between stage 1 and stage 2 were further analyzed. Thirty two DEGs with RPKM >10 were up-regulated 16- to 603-fold and 41 DEGs with RPKM >10 were up-regulated 8- to 16-fold at stage 2. Thirty eight DEGs with RPKM >10 were down-regulated 16- to 89-fold and 41 DEGs were down-regulated 8- to 16-fold at stage 2. These DEGs can be clustered into 11 functional categories as detailed below.

### Auxin homeostasis and signaling

During stage 1, 17 genes including five IAA-amido synthetase *GH3* genes (Vr39322, Vr42095, Vr13365, Vr41585, and Vr13365), ten auxin-induced protein genes, and two auxin-responsive protein genes (Vr44933 and Vr16403) were strongly up-regulated; only an IAA-amino acid hydrolase ILR1-like and an auxin-induced protein 5NG4-like gene were down-regulated. During stage 2, 11 genes including five *GH3* genes, four auxin-induced protein genes, an auxin-responsive protein IAA12-like gene, and an auxin-binding protein ABP19a-like gene were up-regulated; the hydrolase activity gene was down-regulated. When compared with stage 1, the gene encoding for auxin-binding protein ABP19a-like (RPKM =52.8) was specifically up-regulated at stage 2. Sixteen auxin-induced protein genes were down-regulated. The *GH3* gene family encodes IAA-amido synthetases that negatively regulate the levels of free IAA by conjugating IAA to amino acids. IAA-amino acid hydrolase hydrolyzes the amide bond between IAA and the conjugated amino acid to release active IAA. IAA-amino acid hydrolase coordinates with PIN and GH3s to maintain auxin homeostasis in the adventitious roots [7]. The IBA-induced overexpression of the *GH3* genes and the IBA-mediated repression of the IAA-amino acid hydrolase gene both in stage 1 and stage 2 suggest a reduction of free auxin and an increase in amino-IAA during the early stages of adventitious rooting. The auxin-induced overexpression of the *GH3-3* gene was also investigated during the initiation of adventitious roots in both the hypocotyls and stems of *Arabidopsis* [52]. In addition, GH3s also control jasmonic acid (JA) homeostasis by conjugating JA to amino acids. Auxin controls JA homeostasis and stimulates adventitious rooting by inducing *GH3* expression, leading to an increase in JA conjugation and a reduction in free JA levels. This signaling pathway fine-tunes adventitious root initiation in *Arabidopsis* hypocotyls [13]. An additional auxin receptor, ABP1 (AUXIN BINDING PROTEIN1), rapidly mediates cellular auxin effects via a non-transcriptional auxin response pathway and is essential for early auxin responses [53]. The specifically up-regulated ABP19a-like gene indicates that the mediation of ABP in rapid auxin signaling occurred in stage 2. Moreover, genes encoding auxin-induced proteins were down-regulated in stage 2, which may result in the initiation of auxin signaling due to the derepression of ARFs during stage 2.

### Ethylene pathway-related DEGs

During stage 1, eight genes, including three 1-aminocyclopropane-1-carboxylic acid (ACC) synthase (ACS) genes (Vr45385, Vr10942, and Vr10988), three ethylene-responsive transcription factor 1B-like genes (Vr28889, Vr42199, and Vr30921), a S-adenosylmethionine (SAM) decarboxylase proenzyme-like gene, and a L,L-diaminopimelate aminotransferase gene, were up-regulated; an ACC oxidase (ACO) homolog 1-like gene was down-regulated. No DEGs were selected with the setting criteria during stage 2. When compared with stage 1, the genes for an ethylene-responsive transcription factor LEP-like and an ethylene receptor were up-regulated; genes coding for an ACS, two ACO (Vr43821 and Vr44578), two ethylene-responsive transcription factor 15-like (Vr31499 and Vr52928), and a L,L-diaminopimelate aminotransferase were down-regulated. S-adenosylmethionine decarboxylase and L,L-diaminopimelate aminotransferase are involved in ethylene biosynthesis [54]. In the ethylene biosynthetic pathway, ACS converts SAM to ACC, and then ACO converts ACC to ethylene [54]. This result indicates that IBA significantly up-regulates the expression of *ACS* and subsequently promotes ethylene biosynthesis [6, 55]. Ethylene biosynthesis and perception and ethylene–auxin crosstalk are required for adventitious root formation [56]. Auxin promotes ethylene biosynthesis by up-regulating the expression of several *ACS* genes [54], thereby increasing ethylene levels [56]. Ethylene positively regulates IAA synthesis and promotes polar auxin transport and accumulation via AUX1 [55–57].

### Cytokinin pathway-related DEGs

Several genes involved in the cytokinin biosynthesis pathway, including two cytokinin riboside 5’-monophosphate phosphoribohydrolase genes (Vr51879 and Vr25044) and a cytokinin dehydrogenase 3-like gene, were down-regulated during stage 2 compared with stage 1. Cytokinin has been known to be a negative regulator of adventitious rooting via its negative regulation of auxin [25]. IBA-induced adventitious rooting might be related to the inhibition of cytokinin synthesis by IBA in this study.

### Transcription factors

During stage 1, nine genes including a LOB29 gene, a LOB16 gene, two protein SOMBRERO-like genes (Vr44306 and Vr49314), three homeobox-leucine zipper protein ATHB-like genes (Vr74958, Vr21565 and Vr33455), a WRKY (WRKYGQK domain) 72-like gene, and a bHLH (basic/helix-loop-helix) 135-like gene were highly up-regulated; six genes including a MYB1(avian myeloblastosis viral oncogene homolog) 13-like gene, a MYB-related protein 308-like gene, a bHLH18-like gene, a transcription factor CPC gene, and a LOB 21-like gene were down-regulated. During stage 2, genes encoding LOB domain proteins such as LOB 12-like, LOB 16-like, LOB 18-like, and LOB 29-like were up-regulated; a MYB113-like gene was down-regulated. When compared with stage 1, genes coding for a SCR (SCARECROW)-like protein 28-like and a MADS-box transcription factor 1-like were up-regulated, and genes coding for bHLH137-like, BEE 3-like, and NAC (NAM, ATAF, and CUC2) 72-like were down-regulated at stage 2. SOMBRERO-like genes encoding NAM (NO APICAL MERISTEM) family proteins modulate meristems and primordial development and promote the expression of genes involved in secondary cell walls biosynthesis [58]. *SCR* genes in the GRAS family are known to be involved in meristem maintenance and root meristem initiation in adventitious root primordia [24, 59, 60]. Interestingly, the expression of these genes is induced by exogenous auxin in the rooting-competent cuttings of two distantly related forest species, *Pinus radiata* and *Castanea sativa*, during the early stage leading to adventitious root formation [24].

### Cell wall modification

During stage 1, seven genes including two expansin precursor genes (Vr38364 and Vr49341), two UDP-glucosyltransferase genes (Vr236166 and Vr36946), a nonsymbiotic hemoglobin gene, a cationic peroxidase 1-like gene, and a cytokinin induced message gene were up-regulated; 15 genes including three genes of fasciclin-like arabinogalactan protein (Vr36181, Vr22704, and Vr40615), five genes encoding cellulose synthase (Vr46819, Vr44425, Vr43928, Vr41179, and Vr38347), three genes encoding glycosyl transferase family protein (Vr39089, Vr36076, and Vr48851), a COBRA-like protein gene, an organ-specific protein S2-like gene, a lectin gene, and a polygalacturonase non-catalytic subunit JP650-like gene were down-regulated. Expansins have been demonstrated to be induced in hypocotyl bases during the early stages of adventitious root induction in *Pinus taeda* and function in the loosening of cellulose and hemicellulose [61]. The cytokinin-induced message protein, which belongs to expansin family protein, is considered to be involved in cell wall loosening [58]. Cationic peroxidase has been shown to be involved in the biosynthesis of lignin and suberin [62]. Polygalacturonase is one of the pectin lyase associated with the degradation of the pectin network in the plant cell wall [58]. The ortholog of fasciclin-like arabinogalactan protein of *Arabidopsis* may be a cell surface adhesion protein that is required for normal cell expansion and is involved in cell wall biogenesis and organization [58]. COBRA-like protein is involved in cellulose microfibril organization [58]. Therefore, our results indicate that the process of cell wall loosening was significantly up-regulated, whereas the process of cell wall synthesis was suppressed by IBA after 6 h of treatment.

During stage 2, 10 genes associated with cell wall loosening including two expansin genes (Vr55545 and Vr38364), two protein SOMBRERO-like genes (Vr44306 and Vr49314), a galactoside 2-alpha-L-fucosyltransferase-like, a polygalacturonase-like, a pectate lyase 8-like, a cytokinin-induced message, two cationic peroxidase 1-like genes (Vr43362 and Vr46509), and a BURP domain-containing protein 3-like were up-regulated; 17 genes associated with cell wall synthesis including six cellulose synthase A catalytic subunit 7 genes (Vr54568, Vr43928, Vr41179, Vr44425, Vr46089, and Vr46819), four genes encoding casparian strip membrane protein (CASP) (Vr36698, Vr68124, Vr79590, and Vr77808), three fasciclin-like arabinogalactan protein genes (Vr40615, Vr37484, and Vr36181), a xylosyltransferase 1-like gene, a lectin gene, an organ-specific protein S2-like gene, a xyloglucan endotransglucosylase/hydrolase protein 32-like genes, a UDP-galactose transporter 2-like gene, a COBRA-like protein 4-like gene, a laccase-3-like gene, and a mannan endo-1,4-beta-mannosidase 4-like gene were down-regulated during stage 2.

When compared with stage 1, putative expansin-A17-like (RPKM =43.6) was specifically up-regulated at stage 2. Seventeen genes including three polygalacturonase PG1 genes (Vr39755, Vr41762, and Vr44288), two endoglucanase (Vr34411 and Vr53145), a galactoside 2-alpha-L-fucosyltransferase-like gene, two UDP-glucosyltransferase family protein (Vr36280 and Vr39089), an acidic endochitinase, a rhamnose biosynthetic enzyme 1-like, a soyasaponin III rhamnosyltransferase, two glucan endo-1,3-beta-glucosidase-like (Vr43285 and Vr18182), and two extensin-like proteins (Vr58311 and Vr46239) were up-regulated; nine genes including a lignin-forming anionic peroxidase-like, two xyloglucan endotransglucosylase/hydrolase (Vr34550 and Vr39412), two UDP-glycosyltransferase 83A1-like (Vr45972 and Vr47846), a beta-glucosidase 47-like, a cellulose synthase-like protein E1-like, an expansin-like B1-like, and an acetyltransferase gene were down-regulated.

Of these, SOMBRERO-like genes are involved in secondary cell wall biogenesis [58]. BURP domain-containing protein 3-like has polygalacturonase activity that is involved in auxin polar transport and cell wall organization [58]. The gene laccase-3-like is involved in the lignin degradation and detoxification of lignin-derived products [58]. Casparian strip membrane proteins (CASPLs) have recently been shown to form membrane scaffolds and direct the local modification of the cell wall in *Arabidopsis thaliana* [63]. The gene galactose oxidase-like is involved in lignin degradation pathways. The snakin-1-like protein and acidic endochitinase are members of the glycoside hydrolase (GH) family and potentially participate in cellulose catabolic processes and cell wall loosening [64]. The galactoside 2-alpha-L-fucosyltransferase-like gene is involved in cell wall biogenesis. Extensin-like protein is a member of the leucine-rich repeat (LRR) family protein and serves as structural constituent of the cell wall [65]. Acetyltransferase plays a role in the cutin biosynthetic process [58]. In summary, these results suggest that most of the genes associated with cell wall loosening were strongly induced by IBA at stage 1, while genes with the potential to be active in cell wall synthesis were repressed by IBA in stage 1. Conversely, the majority of genes involved in cell wall weakening began to be down-regulated and most of the genes participating in cell wall synthesis were highly up-regulated by IBA in stage 2. These results suggest that one of the mechanisms by which IBA promotes adventitious rooting is the effective regulation of the expression of genes associated with cell wall loosening and remodeling.

### Cell redox homeostasis and stress response

During stage 1, two glutathione S-transferase (GST) genes (Vr42761 and Vr40194), a cationic peroxidase 1-like gene, a nitronate monooxygenase-like gene, three seed maturation protein LEA 4 genes (Vr18423, Vr12933, and Vr23198), a low-temperature-induced 65-kDa protein-like gene, a cytochrome P450 82A3 gene, a 2-oxoglutarate/Fe(II)-dependent dioxygenase gene, and a probable nucleoredoxin 2-like isoform 2 were up-regulated; four genes encoding lipoxygenases (Vr38595, Vr25042, Vr40522, and Vr43523), a protein IQ-DOMAIN 1-like gene, and a cytochrome P450 monooxygenase CYP93D1 gene were down-regulated. During stage 2, three peroxidase 1-like genes (Vr43362, Vr46509, and Vr40373), a FAD-linked oxidoreductase 1 gene, and a gibberellin 3-beta-dioxygenase 3-like gene as well as genes responding to water deprivation such as a lachrymatory-factor synthase-like, a potassium channel AKT1-like, and a DNA-damage-repair/toleration protein DRT100-like precursor gene were up-regulated; five lipoxygenase genes (Vr25042, Vr42442, Vr43510, Vr43523, and Vr38595), an early nodulin-like protein 1-like (electron carrier activity) gene, three peroxidase genes (Vr46854, Vr45676, and Vr28565), three protein SRG1-like (iron/ascorbate family of oxidoreductases) genes (Vr53443, Vr26676, and Vr13190), and two protein IQ-DOMAIN 1-like genes (Vr12929 and Vr49621) were down-regulated. When stage 2 was compared with stage 1, eight peroxidase genes (Vr43362, Vr41032, Vr42734, Vr48815, Vr59402, Vr67115, Vr31903, and Vr40216) and three cationic peroxidase 1-like genes (Vr43362, Vr46509, and Vr58732) were up-regulated; a 2-oxoglutarate/Fe(II)-dependent dioxygenase-like, two cytochrome P450 (Vr37396 and Vr45214), two peroxidase genes (Vr39358 and Vr40642), a GIR1, protein IQ-DOMAIN 14-like, two pleiotropic drug resistance protein 1-like (an ABC transporter G family member) (Vr32712 and Vr48443), and the embryo-abundant protein EMB (S-adenosylmethionine-dependent methyltransferase) were down-regulated.

Glutathione S-transferases (GSTs) play a vital role in the responses to various stresses and were highly induced by IBA [66]. Peroxidase activity has been shown to clearly increase during IBA-induced adventitious rooting [34]. Lachrymatory-factor synthase-like, a pyrabactin resistance 1-like (PYR1) protein, functions as an abscisic acid sensor and mediates its signaling [67]. Lipoxygenases (LOXs) have been shown to be involved in growth, development and oxidative stress [68] and are significantly expressed in tissue cultures for adventitious rooting [22]. In plants, LOXs oxidize linolenic acid present in membranes to result in the lipoperoxidation of membranes [69, 70]. The cytochrome P450 monooxygenase family of proteins has a variety of functions including anthocyanin accumulation, brassinosteroid biosynthetic processes, cell wall organization, oxidation-reduction processes, and indole-containing compound metabolic processes [71]. A member of cytochrome P450 has been shown to regulate adventitious rooting, and a loss of function mutant for the cytochrome P450 *CYP83B1*, which is involved in the indole glucosinolate pathway, spontaneously produces an excess of adventitious roots [72]. IQ-DOMAIN 1 is a Ca^2+^-dependent calmodulin-binding protein involved in glucosinolate metabolism in response to biotic challenge [73]. GIR1, a member of the GASA gibberellin regulated cysteine rich protein family, is up-regulated by the plant hormone gibberellin and is involved in defense responses [74]. Late embryogenesis abundant (LEA) proteins act as water-binding molecules, membrane-stabilizers, and ion modulators and are induced by drought stress [50, 75]. Although the changes in gene expression in response to IBA are complex, analysis from our results indicates that antioxidative processes and water deprivation responses were highly up-regulated, while lipoperoxidation of membranes were strongly alleviated by IBA treatment during the early stages of adventitious rooting.

### Secondary metabolites and flavonoid and terpenoid biosynthesis

During stage 1, one flavanone 3-dioxygenase-like gene was up-regulated, while 16 genes, including three flavanone 3-hydroxylase genes (Vr39135, Vr67264, and Vr39068), a beta-amyrin synthase gene, a 3-hydroxy-3-methylglutaryl-CoA reductase 1-like gene, an anthocyanin 3’-O-beta-glucosyltransferase-like gene, a ropinone reductase homolog gene, an anthocyanidin synthase gene, a secologanin synthase-like isoform 1 (cytochrome P450) gene, a hydroxymethylglutaryl-CoA synthase-like gene, a UDP-glycose:flavonoid glycosyltransferase gene, a 8-hydroxyquercetin 8-O-methyltransferase-like isoform 1 gene, a hydroxyl cinnamoyl-CoA shikimate/quinate hydroxyl cinnamoyl transferase-like gene, a 1-deoxy-D-xylulose-5-phosphate synthase gene (isopentenyl transferase involved in terpenoid biosynthesis), a polyphenol oxidase gene, a cytochrome b5-like gene, and a protein TRANSPARENT TESTA 12-like gene (a flavonoid biosynthesis pathway gene [58]), were down-regulated. During stage 2, an ent-kaurenoic acid oxidase 2-like gene was up-regulated, while a 1-deoxy-D-xylulose-5-phosphate synthase gene, a chalcone synthase-like (CHS), and a flavanone 3-hydroxylase gene were down-regulated. When compared with stage 1, a UDP-glycose:flavonoid glycosyltransferase, a hydroxycinnamoyltransferase-like (spermidine hydroxy cinnamoyl transferase), a 3-hydroxy-3-methylglutaryl-CoA reductase 1-like (involved in terpenoid backbone biosynthesis), an ent-kaurenoic acid oxidase 2-like, an 7-ethoxycoumarin O-deethylase-like gene (flavonoid 3’-monooxygenase, has oxidoreductase activity), and a 3’-hydroxy-N-methyl-(S)-coclaurine 4’-O-methyltransferase-like (S-adenosylmethionine-dependent methyltransferases) gene were up-regulated; 11 genes including two isoflavone reductase-like (NAD(P)H-dependent oxidoreductase activity) (Vr34208 and Vr33951), three isoflavone synthase (Vr43861, Vr50428, and Vr47942), an isoflavone 2’-hydroxylase-like (cytochrome P450), two leucoanthocyanidin dioxygenase-like (Vr36176 and Vr44347) (involved in anthocyanin-containing compound biosynthetic process), a flavanone 3-dioxygenase-like, and two isoliquiritigenin 2’-O-methyltransferase-like genes (Vr49125 and Vr47010) were down-regulated.

Flavonoid metabolism exerts roles in development, signaling, and stress responses in plants. Flavonoids were shown to be involved in adventitious rooting [22, 31]. In addition, 8-hydroxyquercetin 8-O-methyltransferase, quinate hydroxyl cinnamoyl transferase, and polyphenol oxidase are also involved in the lignin biosynthetic pathway. The present study indicates that IBA treatment principally down-regulated the expression of genes associated with secondary metabolism, including flavonoid synthesis and lignin biosynthesis, during the early stages of adventitious rooting, especially at root induction stage in mung bean seedlings. During the root primordia formation phase in *P. contorta*, transcripts encoding enzymes of the flavonoid pathway were up-regulated [27]. One of these flavonoid pathway proteins, chalcone synthase, and a pathogenesis-related protein contribute to a constitutive defense barrier in the root epidermis of the pea [76].

### Amino acid and nucleotide metabolism and protein transport and degradation

During stage 1, two 5’-adenylylsulfate reductase 3 genes (Vr42149 and Vr44003), a CTP synthase-like, and an adenosine deaminase-like gene were up-regulated; three genes related to protein folding and degradation including two trypsin protease inhibitor genes (Vr34971 and Vr35851), a RING-H2 finger protein ATL80-like, and a chaperonin CPN60-like 2 gene were down-regulated. Nutrient reservoir-related genes such as a stem 28-kDa glycoprotein-like gene, a glutelin type-A 2-like gene, and an ATP-citrate synthase alpha chain protein 1-like gene were down-regulated. During stage 2, a prunin 2 precursor, an oligopeptide transporter 1-like, a RING-H2 finger protein ATL60-like, an aspartic proteinase nepenthesin-1-like, and a vignain-like gene were up-regulated; two chaperonin CPN60-like 2 genes (Vr40776 and Vr24119), an aspartic proteinase-like, a multicystatin, a stem 28-kDa glycoprotein-like, a probable purine permease 9-like, and a cucumisin-like gene were down-regulated.

When stage 2 was compared with stage 1, a prunin 2 precursor, a 34-kDa maturing seed protein, an oligopeptide transporter 1-like, a probable amino acid permease 7-like, a probable E3 ubiquitin-protein ligase HERC1-like gene, two heat shock 70-kDa protein-like (Vr40796 and Vr42894), and an aspartic proteinase nepenthesin-1-like were up-regulated; a germin-like protein 14, a glutamine amidotransferase-like protein, two lysine histidine transporter (Vr55277 and Vr54148), two cysteine protease (Vr38917 Vr38984, and Vr60819), a leurain-like protease, a proton-coupled amino acid transporter, a branched amino acid transaminase, an adenosine deaminase-like protein-like, an aspartic proteinase nepenthesin-1-like, a dnaJ-like protein R260-like, a formate dehydrogenase, an E3 ubiquitin-protein ligase RMA1H1-like, and a probable GMP synthase-like gene were down-regulated.

Of those, 5’-adenylylsulfate reductase 3 reduces sulfate for cysteine biosynthesis involved in cysteine biosynthesis and cell redox homeostasis [58]. Adenosine deaminase-like, which is a putative nucleoside deaminase, may catalyze the hydrolytic deamination of adenosine and play a role in purine metabolism [58]. RING-H2 finger proteins, E3 ubiquitin-protein ligase, and heat shock 70-kDa protein-like are involved in the pathway of protein ubiquitination and in the early steps of the plant defense signaling pathway [58]. Chaperonin CPN60-like 2 may facilitate the correct folding of imported proteins and is implicated in stress response [58]. DnaJ-like protein R260-like also is a chaperone protein, which is required for peroxisomal protein import and maintains the function of peroxisomes [58]. Stem 28-kDa glycoprotein-like, glutelin type-A 2-like, and prunin 2 precursor, and germin-like protein are seed storage proteins. ATP-citrate synthase alpha chain protein 1-like has transferase activity, which transfer acyl groups and is involved in cellular carbohydrate metabolic process [58]. Oligopeptide transporter 1-like is an amino acid/oligopeptide transporter. Aspartic proteinase nepenthesin-1-like, aspartic proteinase-like, vignain-like, cucumisin-like, and 34-kDa maturing seed protein act as an endopeptidase which is involved in mobilizing storage proteins in seeds and response to water deprivation [58]. GMP synthase-like, also glutamine amidotransferase, is involved in glutamine-hydrolyzing. Multicystatin is a cysteine-type endopeptidase inhibitor, which probably has a role in the plant defense system and is induced by wounding [58]. Formate dehydrogenase and E3 ubiquitin-protein ligase RMA1H1-like also are involved in response to wounding. These results indicate that IBA up-regulated the genes associated with the synthesis and transport of amino acids and nucleotides but down-regulated the genes involved in the protein degradation and processing during early stage of rooting. Furthermore, IBA may alleviate the stresses of wounding and water deprivation occurred at the root initiation stage via up-regulating the expression of the genes associated with protein processing, such as the genes of chaperonin and heat shock 70.

### Lipid and sugar metabolic processes

A sterol 4-alpha-methyl-oxidase gene and an omega-6 fatty acid desaturase gene were up-regulated, and a patatin-like protein was down-regulated during stage 1. Four genes coding for patatin group A-3-like (Vr43029, Vr58791, Vr22680, and Vr22680) and a carboxylesterase 15-like gene were down-regulated during stage 2. When compared with stage 1, two GDSL esterase/lipase CPRD49 genes (Vr13168 and Vr29049), a lipid transfer-like protein, and a bidirectional sugar transporter SWEET3-like gene were up-regulated; a sterol 4-alpha-methyl-oxidase was down-regulated.

Sterol 4-alpha-methyl-oxidase and omega-6 fatty acid desaturase are involved in fatty acid biosynthetic process [58]. Omega-6 fatty acid desaturase catalyze the biosynthesis of 18:3 fatty acids, which is an important constituent of plant membranes [58]. GDSL esterase/lipase CPRD49 and carboxylesterase 15-like are the members of esterases and lipases, and patatin-like and patatin group A-3-like are the members of phospholipase, which are involved in lipid degradation and plant defense [58]. Obviously, IBA up-regulated the expression of genes associated with fatty acid synthesis but down-regulated the genes for lipid hydrolization during both stages 1 and 2. Furthermore, IBA up-regulated the genes associated with lipid and sugar transport as well as lipid degradation from stage 1 to stage 2.

### Photosynthesis

Two genes for LHCII type I chlorophyll a/b-binding proteins (Vr22793 and Vr23315) were down-regulated during stage 1. Four LHCII type I chlorophyll a/b-binding protein genes (Vr38329, Vr22793, Vr38285, and Vr22637), a photosystem II 22-kDa protein, and a chlorophyll a-b binding protein gene were up-regulated during stage 2 compared with stage 1, suggesting that photosynthetic activity was reduced during stage 1 and then was increased within 24 h by IBA treatment.

### Signal transduction

A leucine-rich repeat receptor-like protein kinase PXL2-like and a membrane-associated kinase regulator 6-like gene were up-regulated during stage 1. When compared with stage 1, genes encoding a CBL-interacting serine/threonine-protein kinase 21-like, a leucine-rich repeat receptor-like protein kinase IMK2-like, and two protein suppressor of PHYA-105 1-like genes (Vr26363 and Vr48612) were up-regulated; a membrane-associated kinase regulator 6-like gene was down-regulated.

Leucine-rich repeat receptor-like protein kinase PXL2-like, CBL-interacting serine/threonine-protein kinase 21-like, leucine-rich repeat receptor-like protein kinase IMK2-like, and membrane-associated kinase regulator 6-like belong to the transmembrane receptor protein serine/threonine kinase, which is involved in signaling pathway and cell differentiation [58]. Protein suppressor of PHYA-105 1-like is involved in the phytochrome signaling pathway [58]. The result suggests that IBA mediated in the signaling transduction for promoting adventitious rooting.

### Cell cycle

Genes coding for a syntaxin-related protein KNOLLE-like, three G2/mitotic-specific cyclins (Vr45997, Vr48717, and Vr24479), and an anaphase-promoting complex subunit cdc20-like protein were up-regulated during stage 2 compared with stage 1. Syntaxin-related protein KNOLLE-like direct movement of proteins in a cell and the membrane organization process [58]. G2/mitotic-specific cyclins are essential for the control of the cell cycle at the G2/M and G1/S (mitosis) transition [57]. Anaphase-promoting complex subunit cdc20-like is implicated in a variety of functions ranging from signal transduction and transcription regulation to cell cycle control [58]. This indicates that cell mitosis started from the stage 2 and IBA promoted this process.

### Other genes

A gene encoding a soluble inorganic pyrophosphatase-like protein was down-regulated at stage 2. When compared with stage 1, a soluble inorganic pyrophosphatase-like and a nitrate transporter 1.1-like gene were up-regulated; Over-expression of the pyrophosphatase gene increases salt and drought resistance and shoot and root biomass as well as improves nutrient use efficiencies [77]. Nitrate transporter was reported to act as a nitrate sensor that trigger a specific auxin-activated signaling pathway stimulating lateral root growth and may be involved in response to water deprivation [58, 78]. Clearly, our results may indicate that IBA also modulates the phosphate-containing compound metabolic process and nitrate transport to response to certain stresses and further for adventitious rooting.

### Genes involved in plant hormone signaling were significantly regulated by IBA during the adventitious rooting process

Given that plant hormones are generally considered to be vital modulators of adventitious rooting, we further examined the genes coding for proteins involved in plant hormone signaling transduction during stage 1 and stage 2. A total of 143 unigenes (fold change > 2), including 92 auxin-related genes, 37 ethylene-related genes, five cytokinin-related genes, and eight gibberellin genes, were identified (Additional file 7).

In the group of auxin-related genes, a total of 60 genes, with 52 up-regulated 2.2- to 88-fold and eight down-regulated 3- to 8-fold, and a total of 41 genes, with 33 up-regulated 2- to 37-fold and seven down-regulated 2- to 11-fold, were identified at stage 1 and stage 2, respectively. The down-regulated genes at stage 1 included an ABC transporter, two Aux5NG4-like, a PIN4b (auxin efflux carrier), and four IAA-amino acid hydrolase ILR1-like genes; at stage 2, the genes included six Aux5NG4-like genes and an auxin response factor 9-like gene. Compared with stage 1, a total of 38 genes, including 11 up-regulated and 37 down-regulated, were detected at stage 2. The up-regulated genes were primarily members of the ABC transporter G family, while down-regulated genes were mostly members of the AUX family. Auxin efflux carriers control auxin distribution to establish and maintain auxin concentration gradients in various tissues [79], triggering the establishment of new growth axes [80]. The ABC transporter has been known to function as an auxin carrier complex in cellular auxin efflux and influx [81]. These results indicate that most of the genes related to auxin signaling were significantly up-regulated by IBA treatment. The down-regulation of PIN and the ABC transporter during stage 1 and up-regulation of the ABC transporter during stage 2 suggest that auxin transport occurred in stage 2. In other studies, the expression of *PIN1* and *AUX* were up-regulated by IBA treatment and are essential for adventitious root formation [15].

In the group of ethylene-related genes, a total of 17 genes, with 15 up-regulated 2.2- to 42-fold and two down-regulated, and a total of 11 genes, with eight up-regulated 2- to 18-fold and three down-regulated, were identified at stage 1 and stage 2, respectively. Compared with stage 1, there were a total of 20 genes, with 9 up-regulated and 11 down-regulated genes, at stage 2. Genes coding for an ACS and an ethylene receptor were up-regulated both at stage 1 and stage 2; however, genes coding for an ACC oxidase, an ACS, and three AP2/ERFs were down-regulated from stage 1 to stage 2. Ethylene biosynthesis has been demonstrated to be required for adventitious root formation, and there is crosstalk between ethylene and auxin during the process of adventitious root formation [56, 82]. These results suggest that genes associated with ethylene synthesis and signaling were up-regulated by IBA treatment. It appears that IBA-induced ethylene production may be a factor involved in the stimulation of adventitious rooting [11].

Five cytokinin-related genes were detected. Of these, a cytokinin-induced message coding gene was up-regulated both at stage 1 and stage 2, while four genes coding for enzymes involved in cytokinin synthesis were down-regulated at stage 1 and from stage 1 to stage 2. Exogenous application of IBA greatly inhibited genes involved in cytokinin biosynthesis [31].

Eight genes involved in the gibberellin (GA) signaling pathway were detected, among which a gibberellic acid-stimulated protein 1 and a gibberellin 3-beta-dioxygenase 3-like gene were up-regulated both at stage 1 and stage 2 as well as from stage 1 to stage 2, and five gibberellin oxidase (GA20ox) genes and a GIR1 gene were down-regulated from stage 1 to stage 2. Both gibberellic acid-stimulated protein 1 and GIR1 are the GASA gibberellin regulated cysteine rich proteins, which regulate many plant development processes via cell division and/or elongation and respond to biotic and abiotic stresses [58, 74, 83]. Gibberellin 3-beta-dioxygenase 3-like is involved in gibberellin biosynthesis, which converts the inactive GA precursors GA9 and GA20 in the bioactives GA4 and GA1. GA20ox is a key oxidase enzyme in the biosynthesis of GA that catalyzes the conversion of GA12 and GA53 to GA9 and GA20 respectively [58]. GA treatment has been shown to negatively affect adventitious rooting [84]. This study indicates that IBA treatment inhibited the GA synthesis genes, leading to GA synthesis inhibition.

### Transcription factor (TF) encoding genes were significantly regulated by IBA during the process of adventitious rooting

To further understand the roles of transcription factors in regulating the early stages of rooting, the expression levels of a total of 1,008 TF coding genes that were differentially expressed in the samples were analyzed. Among these, 345 TF genes were significantly expressed with fold-changes >2, of which 204 TF genes with 107 up-regulated and 97 down-regulated were detected at stage 1, 199 TF genes with 38 up-regulated and 161 down-regulated were detected at stage 2, and 204 TF genes with 77 up-regulated and 127 down-regulated were detected in stage 2 versus stage 1. The majority were from TF families such as auxin-response factors (ARF, 12), indoleacetic acid-induced protein (AUX/IAA, 7), LOB (7), MYB (32), MYC (3), bHLH (31), WRKY (18), NAC (25), APETALA2 and ethylene-responsive element binding proteins (AP2/ERF, 31), HSF (10), homeobox leucine protein (24), zinc finger protein (33), DOF (8), basic leucine zipper (bZIP, 7), C2H2 (3), and E2F (2).

The most differentially expressed TF genes are summarized in Additional file 8. Three NAM family proteins, specifically two protein SOMBRERO-like and a protein FEZ-like, which modulates meristems and primordial development, were up-regulated by approximately 16- to 32-fold at stage 1 and stage 2. In the LOB family, *LOB16* and *LOB29* were up-regulated approximately 16- to 32-fold both at stage 1 and stage 2, while *LOB4* and *LOB21* were down-regulated by approximately 2- to 16-fold both at stage 1 and stage 2. This result indicates that, in the LOB family, *LOB16* and *LOB29* were highly up-regulated by IBA treatment. A number of auxin-responsive LOB-domain genes have been found to act early in auxin signaling and regulate adventitious rooting [18]. Of these, the *Arabidopsis* genes *LBD16* and *LBD29* were up-regulated by auxin and directly activated by the products of the early auxin-responsive genes *ARF7* and *ARF19* [85, 86]. LBD29 maintains the cell division capacity of the pericycle in response to auxin [87].

The AP2/ERF, MYB, NAC, WRKY, and bHLH families were found to be significantly expressed during early stages of adventitious root formation in poplar, and the MYB and AP2/ERF families were the most highly modulated transcription factors [26]. Among the six *AP/ERF* genes detected, an ethylene-responsive transcription factor 4-like was up-regulated at stage 1 and five genes were down-regulated at stage 2. AP2/ERFs have been shown to regulate a number of developmental processes [88]. AP2/ERF families were the most highly modulated transcription factors during IBA-induced adventitious rooting [26, 89]. They have been shown to be key endogenous regulators of adventitious rooting in poplar [90].

In the bHLH family, two genes (Vr34156 and Vr33273) were up-regulated at stage 1 and two genes (Vr44886 and Vr26937) were up-regulated at stage 2 and from stage 1 to stage 2; however, *bHLH137* was down-regulated at stage 2 and from stage 1 to stage 2. In the MYB family, *MYB134* was down-regulated at stage 1 and stage 2 but up-regulated from stage 1 to stage 2. *MYB308*, *MYB363*, *MYB113*, and *MYB114* were down-regulated at stage 1 or stage 2 but up-regulated from stage 1 to stage 2. *MYB315* was down-regulated at stage 2 and from stage 1 to stage 2. In the NAC family, six NAC domain protein coding genes (Vr40604, Vr21172, Vr52381, Vr42870, Vr42963, and Vr50705) were down-regulated at stage 1 and stage 2 with the exception of Vr25638, which was up-regulated from stage 1 to stage 2. Eight zinc finger protein genes were down-regulated at stage 1 or stage 2 and from stage 1 to stage 2. In addition, a *WRKY 2* gene was down-regulated 32-fold at stage 1. A WUSCHEL-related homeobox (*WOX*) gene, which encodes an auxin-inducible transcription factor that is highly responsive to auxin and is specifically expressed in quiescent center (QC) maintenance and the root apical meristem (RAM) [91], was up-regulated at stage 2, suggesting the occurrence of RAM formation in stage 2. These results indicate that the majority of bHLH transcription factors were up-regulated, while the majority of MYBs, NACs, zinc finger proteins, and WRKY were down-regulated by IBA treatment during the early stages of adventitious rooting.

### Validation of gene expression using qRT-PCR

**Fig. 6:**
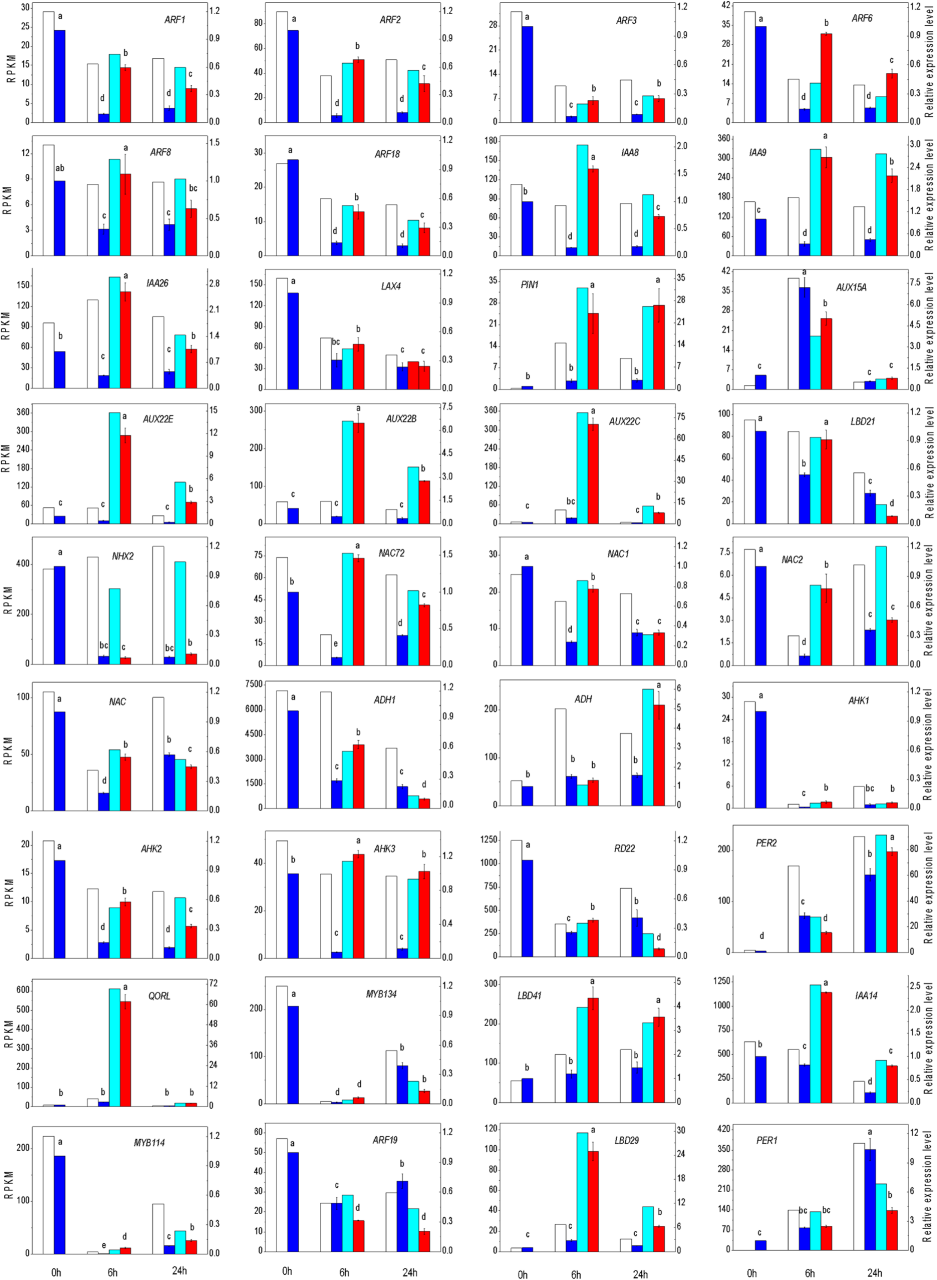
Validation of selected genes involved in adventitious rooting by qRT-PCR. The gene expression levels measured by qRT-PCR were compared with that of RNA-Seq. The data of Con, Wat 6, and Wat 24 in the figure are the same as our previous study published recently [39]. White histograms represent expression levels determined by RNA-Seq in RPKM units (left axis), while gray columns represent gene expression levels determined by qRT-PCR and normalized to control genes (right axis). Bars represent the mean (± SE) of three experiments. Different letters (a, b, and c) represent statistically significant differences (*P* < 0.01) among the qRT-PCR data, analyzed using Student’s t-test

To validate the differential expression data obtained through statistical comparison of RPKM values, a total of 36 interesting DEGs of five types were identified: 17 auxin signaling-related genes, 14 stress response-related genes, three *LBD* genes, and two *MYB* genes. These genes were selected for validation of the transcriptomic data using real-time quantitative PCR (qRT-PCR). Detailed information on these genes is presented in Additional file 7. Based on the RNA-Seq results and the study published by Jian et al. [92], we selected three genes, *CPY20*, *eIF*5*A*, and *ACTIN* (*Actin-related protein 4*), as internal reference genes for qRT-PCR experiments. The qRT-PCR results showed that *CPY20* was the most stable housekeeping gene, so it was used to calculate the relative expression levels in this study. The expression levels of all the genes measured by qRT-PCR showed a strong correlation to the RNA-Seq data (Fig. 6). The correlation analysis between the RNA-Seq and qRT-PCR data showed that the correlation coefficient of 29 of the 36 data sets is r > 0.938. During two time points for adventitious rooting, the expression levels of seven *ARF* family genes exhibited significantly decreasing trends both in the water and IBA treatments, but they were significantly up-regulated by IBA, with the exception of *ARF3* and *ARF19*, compared with the controls. The four *IAA* (auxin inducible protein) family genes, four *AUX* family genes, and one *PIN* gene were significantly up-regulated at 6 h both in the water and IBA treatments and were significantly induced by IBA. The *LBD29* and *LBD41* genes were significantly up-regulated by IBA at two time points. The two genes *MYB134* and *MYB114* exhibited sharp decreases at two time points and a slight increase at 24 h compared with 6 h, but their expression levels were up-regulated by IBA at 6 h. The four *NAC* family genes were significantly up-regulated by IBA at 6 h compared with the controls. The genes *AHK2* (*Arabidopsis* histidine kinase) and *AHK3* were significantly up-regulated by IBA at two time points. The genes cationic peroxidase 1 (*PER1*) and *PER2* exhibited large increases at two time points but were less affected by IBA treatment. The gene quinone oxidoreductase-like protein (*QORL*) was strongly up-regulated by IBA at 6 h. The ARF transcription factors mediate auxin signaling at the transcriptional level by regulating the expression of auxin-responsive genes. The *LBD* genes contain at least one auxin-responsive element (AuxRE) and are involved in auxin-mediating development processes. Several members of the LOB-domain transcription factors have been identified and shown to mediate adventitious rooting [19, 22, 52]. Both *LBD16* and *LBD29* were up-regulated by auxin during adventitious rooting in *Arabidopsis* hypocotyls and stems [52]. The *AUX* family genes have also been shown to be essential for adventitious root formation [15]. NAC transcription factors have been reported to contribute to various developmental processes, such as shoot apical meristem (SAM) development [93], lateral root development [94], and secondary wall formation [95]. Moreover, NAC genes are also involved in responses to stress, such as drought and high salinity [96]. AHK1, AHK2, and AHK3 have been identified as cytokinin receptors and are involved in the water stress response during the early vegetative stages of plant growth and regulation of meristem development [97]. *Arabidopsis* mutants lacking AHK2, AHK3, and AHK4 exhibit enhanced adventitious root growth [98]. The quinone oxidoreductase-like protein (*QORL*), also known as NADH dehydrogenase, which is a zinc-binding dehydrogenase, is involved in the electron transport chain, photorespiration, and redox process. Therefore, our results confirmed that IBA can not only enhance the expression of several auxin signaling control genes such as *ARFs*, *AUX/IAAs*, and *PINs*, as well as several genes from the *LOB* and *MYB* transcription factor families, but can promote the expression of various stress response-related genes such as NAC, AHKs, and QORL to further increase the stress tolerance of the cells, thus leading to efficient adventitious rooting.

## Conclusions

This study provides a comprehensive transcriptome dataset for adventitious rooting in mung bean using the NGS-based Illumina paired-end sequencing platform. A comparative analysis of expression profiles between different rooting developmental time-points as well as between water treatment and IBA treatment yielded subsets of DEGs. Functional categories of significantly enriched GO terms and over-represented biological pathways are presented here. Interestingly, DEG analysis revealed the most significantly and abundantly expressed genes in response to IBA, including genes associated with auxin homeostasis and signaling, ethylene pathway, cytokinin pathway, transcription factors, cell wall modification, cell redox homeostasis and the stress response, secondary metabolites and flavonoid and terpenoid biosynthesis, amino acid and nucleotide metabolism and protein transport and degradation, lipid and sugar metabolic process, photosynthesis, signal transduction, and the cell cycle. Furthermore, a total of 143 DEGs specifically involved in plant hormone signaling and a total of 345 TF genes also responded to IBA. The transcriptomic data reveal that profound cellular and metabolic reorganization occurs during the root induction phase. In conclusion, the transcriptomic data generated in the present study for mung bean adventitious rooting provide insights into the molecular mechanisms that control IBA-induced adventitious rooting.

## Materials and methods

### Plant material and culture conditions

Mung bean [*Vigna radiata* (L.) R. Wilczek] seeds were washed in distilled water, surface-sterilized in a 6 % NaClO solution for 15 min and rinsed three times in sterile distilled water. The seeds were subsequently germinated under a thin layer of sterilized perlite in Petri dishes in a growth chamber at 25 ± 1 °C for 36 h in the dark and then at 25 ± 1 °C with a 14-h light/10-h dark photoperiod under white fluorescent lamps (PAR of 100 μM m^−2^ s^−1^). After five days of growth, seedlings that were 5 cm in height were used for the experiments. The primary roots of the seedlings were removed from the bases of the hypocotyls, and the resulting seedlings were incubated in 50-mL beakers (10 per beaker), each containing 40 mL of sterilized distilled water or 10 μM IBA for 6 to 24 h under the same aseptic conditions applied to the seedling culture. The 5-mm-long basal region of each hypocotyl, where adventitious roots develop *in vitro*, was cut and collected separately at the 0, 6, and 24 h incubation time points, and the harvested samples were separately designated as Con, Wat6, IBA6, Wat24, and IBA24 (Fig. 1). Three biological replicates, each consisting of ten stem cutting bases, were collected per treatment and time point, immediately frozen in liquid nitrogen and stored at -80 °C until further analysis.

### Total RNA extraction, cDNA library preparation and Illumina sequencing

Approximately 50 mg of tissue that was fully ground in liquid nitrogen was mixed with 600 μL of buffer Rlysis-P (from kit SK8631, Sangon, Shanghai, China) in a 1.5-mL RNase-free tube for 5 min in a water bath at 65 °C to ensure sufficient lysis. Next, 60 μL of buffer PCA (from kit SK8631) was added and mixed thoroughly, and the mixture was incubated at -20 °C for 3 min. After the mixture was centrifuged at 10,000 × *g* for 5 min at 4 °C, an equal volume of cooled phenol chloroform (phenol water) was added to the supernatant, mixed, and then centrifuged at 12,000 × *g* for 5 min at 4 °C. An equal volume of cooled chloroform was added to the supernatant and mixed. After the sample was centrifuged at 12,000 × *g* for 5 min at 4 °C, an equal volume of cooled isopropanol was added to the supernatant, shaken gently, and left to precipitate for 10 min. After the sample was centrifuged at 12,000 × *g* for 20 min at 4 °C, the pellet was recovered, washed twice with 75 % ethanol, dried for 5–15 min at ambient temperature, dissolved in 50 μL of RNase-free water, and stored at -80 °C. The resulting RNA integrity was quantified with RNA Integrity Number (RIN) values of 8.1-9.9 using a 2100 Bioanalyzer (Agilent Technologies, Santa Clara, CA, USA), and RNA concentration was determined using a NanoDrop ND-1000 Spectrophotometer.

Poly (A) mRNAs were enriched from the equal amounts of total RNA from each sample using Oligo(dT) 25 beads (Invitrogen) according to the manufacturer’s protocol. The purified mRNA was fragmented using the Fragment Mix reactive system at 94 °C for 4 min. The reaction system for first-strand cDNA synthesis was composed of Superscript II reverse transcriptase (Invitrogen), First Strand Master Mix, random hexamer primers, and the fragmented mRNA templates. The reaction conditions were as follows: 25 °C for 10 min, 42 °C for 50 min, and 70 °C for 15 min, with a final hold at 4 °C. Second-strand cDNA was synthesized using Second Strand Master Mix (Invitrogen). After synthesis, the dscDNA fragments were purified with Agencourt AMPure XP Beads (Agencourt), and the 3’ ends were repaired using End Repair Control, followed by purification with AMPure XP beads. Subsequently, Klenow exo (M0212L, NEB) was used to perform adenylation of the 3’ ends of the cDNA fragments. After end repair and A-tailing, Illumina paired-end adapters were ligated to the cDNA fragments using T4 Ligase (Fermentas) and purified twice with AMPure XP Beads. Last, selective PCR was used to enrich and amplify the ligated cDNA for cDNA library construction. The PCR procedure was performed as follows: 98 °C for 30 s; 15 cycles of 98 °C for 10 s, 60 °C for 30 s, 72 °C for 30 s, and 72 °C for 5 min; holding at 4 °C, followed by purification with AMPure XP beads. The quality and quantity of the cDNA library were confirmed using an Agilent 2100 Bioanalyzer and Qubit 2.0 (Life Technologies). Paired-end sequencing of the constructed cDNA library was performed at Sangon Biotech. Co. Ltd. (Shanghai, China) on an Illumina HiSeq 2000 system (Illumina).

### *De novo* assembly and sequence clustering

After the raw sequence processing, the clean reads were *de novo* assembled using the Trinity paired-end assembly method [41]. The assembled sequences were clustered with Chrysalis. The longest sequences that could not be extended on either end within each clustered loci were obtained and defined as unigenes. The unigenes were subjected to similarity alignment against protein and nucleotide sequence databases using BLASTx locally installed BLAST+ v2.2.27 software [99] and MEGABLAST, respectively, at an e-value cut-off of e-5. BLAST annotations were filtered using either subject or query coverage (>30 %) and sequence identity (>50 % for MEGABLAST and >30 % for BLASTx). The assembled unigenes were deposited in the Transcriptome Shotgun Assembly Sequence Database (http://www.ncbi.nlm.nih.gov/sra/SRR1653637) at DDBJ/EMBL/GenBank under the sequence read archive SRR 1653637 and the accession numbers GBXO01000001-GBXO01078617.

### Mapping reads, expression analysis and DEG confirmation

Due to the lack of a reference sequence, the assembled transcripts were assumed the reference sequence. To compute unigene expression levels, the sequences were aligned against the reference transcript sequences [40, 100, 101]. The expression levels of unigenes were measured by mapping back the number of clean reads to the assembled unigenes using BWA-0.6.2-http://bio-bwa.sourceforge.net/ in the end-to-end alignment mode [102]. The number of clean reads mapped to each unigene was calculated and then normalized to RPKM (reads per kb per million reads) using ERANGE3.1 software [30]. Unigene expression levels were analyzed using the DEGseq R package [51] with the MARS (MA-plot-based method with Random Sampling) model. The DEGs between each pair of samples were screened using the Audic-Claverie algorithm [103] with an FDR threshold of ≤0.001 and an absolute value of log2 ≥ 1. Multiple test corrections for the p-value and FDR were performed with the Benjamini-Hochberg correction [104]. To gain insight into the differential expression of genes specifically induced by IBA at each time point, the analyses were performed between IBA6 and Wat6 and between IBA24 and Wat24, as well as IBA24 relative to IBA6, and were represented as stage 1, stage 2, and stage 1 to stage 2, respectively.

### Functional annotation and classification

All resulting unigenes were annotated according to their sequence similarity to previously annotated genes. First, the unigenes were aligned to the public protein databases NR, SWISS-PROT, TrEMBL, Pfam, and CDD using BLASTx with similarity set at >30 % and an E-value ≤ 1e-5. The KOG (Clusters of Orthologous Groups for eukaryotic complete genomes) and KEGG (Kyoto Encyclopedia of Genes and Genomes) pathway annotations were performed by sequence comparisons against the two databases using BLASTALL and KAAS software (ftp://ftp.ncbi.nih.gov/blast/executables/release/2.2.18/) with an E-value ≤ 1e-5. The resulting blast hits were processed using Blast2GO software (version 2.3.5, http://www.blast2go.de/) [41] with an E-value threshold of 1e-5 to retrieve associated GO terms. WEGO software was used for achievement of GO classification [41]. The results that presented the best alignment were used to identify the sequence direction and to predict the coding regions using BLASTx searches against protein databases, with the priority order of NR, SWISS-PROT, KEGG and KOG if conflicting results were obtained. The ESTScan software [105] was used to analyze the unigenes that did not align to any of the above databases. KEGG mapping was used to determine the metabolic pathways. KEGG pathways were retrieved from the KEGG web server (http://www.genome.jp/kegg/) [106–108]. To further enrich the pathway annotations, unigenes were submitted to the KEGG Automatic Annotation Server (KAAS) [43], and the single-directional best-hit information method was selected. To identify the enriched pathways, the phyper test was used to measure the relative coverage of the annotated KEGG orthologous groups of a pathway against the transcriptome background, and the pathways with a p-value ≤ 0.05 were classified as enriched.

### Quantitative reverse transcription PCR validation

To validate the RNA-Seq data, we selected 36 genes that were shown to be involved in adventitious root development for q-PCR analysis. PCR samples were harvested from the hypocotyls of three biological replicates under the same conditions as the samples subjected to Illumina sequencing. Total RNA was extracted using TRIzol reagent (Invitrogen, Carlsbad, CA, USA) and purified on RNeasy mini spin columns (Qiagen) with on-column DNase I treatment according to the manufacturer’s protocol. RNA integrity was examined with an Agilent Bioanalyzer 2100 (Agilent Technologies). First-strand cDNA was synthesized using an AMV First Strand cDNA Synthesis Kit (Roche Applied Science, Mannheim, Germany) according to the manufacturer’s instructions. The gene-specific primer pairs were designed using Primer Premier 5.0 software (Applied Biosystems, Foster City, CA, USA) according to the confirmed sequences (Additional file 9). Real-time PCR was run using a LightCycler 480 II (Roche Applied Science) and ABI SYBR Green PCR Master Mix (ABI, Foster, USA). Reactions were subjected to the following conditions: 95 °C for 3 min followed by 40 cycles of 95 °C for 15 s and 60 °C for 40 s. A melting curve analysis was conducted to evaluate the primer specificity for each primer set to verify the presence of a single melting peak after amplification. ‘No cDNA’ samples (water) and ‘no RT’ samples were included as negative controls. All reactions were performed with three independent biological replicates, and the expression levels calculated for each sample were based on three technical replicates. Output data were generated with Sequence Detector version 1.3.1 software (ABI). The relative expression levels of the selected genes were calculated in relation to the reference gene using the comparative threshold cycle method with the delta-delta Ct method [109]. Statistical analyses were conducted with Student’s t-tests at the *P* < 0.05 level of significance. The expression levels of all the genes measured by qRT-PCR showed a strong correlation to the RNA-Seq data.

## Availability of data and materials

The unigene sequence information of this article is available in the Transcriptome Shotgun Assembly Sequence Database (http://www.ncbi.nlm.nih.gov/sra/SRR1653637) at DDBJ/EMBL/GenBank under the sequence read archive SRR 1653637 and the accession numbers GBXO01000001-GBXO01078617. The datasets supporting the conclusions of this article are included within the article and its additional files.

## Additional files

Additional file 1:
**Sequencing data information.** (DOCX 13 kb) Additional file 2:
**Top list of significantly up- and down-regulated GOs.** (XLSX 43 kb) Additional file 3:
**Top list of significantly up- and down-regulated KOs.** (XLSX 14 kb) Additional file 4:
**Top up- and down regulated DEGs in the sample treated with IBA for 6 h.** (XLSX 18 kb) Additional file 5:
**Top up- and down regulated DEGs in the sample treated with IBA for 24 h.** (XLSX 19 kb) Additional file 6:
**Top up- and down regulated DEGs in IBA24 versus IBA6.** (XLSX 28 kb) Additional file 7:
**Top significantly up- and down- regulated DEGs involved in plant hormone signaling between the samples.** (XLSX 16 kb) Additional file 8:
**Top most differentially expressed transcription factor (TF) coding genes between the samples.** (XLSX 12 kb) Additional file 9:
**List of the genes and primers selected for q-PCR validation.** (DOCX 22 kb)

## Abbreviations

ABCB/PGP: ATP binding cassette-type B/ P-Glycoproteins
ACC: 1-Aminocyclopropane-1-carboxylic acid
ACO: 1-Aminocyclopropane-1-carboxylic acid oxidase
ACS: 1-Aminocyclopropane-1-carboxylic acid synthase
AHK: Arabidopsis histidine kinase
AP2/ERF: Apetala2/Ethylene response factor
ARF: Auxin response factor
ARL1: Adventitious rootiless1
AUX1/LAX: Auxin/IAA
bHLH: Basic/helix-loop-helix
bZIP: basic leucine zipper
CASPL: Casparian strip membrane protein
CHS: Chalcone synthase
CRL1: Crown rootless1
DEGs: Differentially expressed genes
GH: Glycoside hydrolase
GH3: Gretchen hagen 3
GO: Gene ontology
GST: Glutathione S-transferase
IBA: Indole-3-butyric acid
JA: Jasmonic acid
KAAS: Automatic annotation server
KEGG: Kyoto encyclopedia of genes and genomes
KOG: Clusters of orthologous groups for eukaryotic complete genomes
LBD16: Lateral organ boundaries-domain
LEA: Late embryogenesis abundant
LOB-domain: Lateral organ boundaries-domain
LOXs: Lipoxygenases
LRR: Leucine-rich repeat
NAC: NAM, ATAF, and CUC2
NAM: No apical meristem
NR: NCBI non-redundant protein
PER: Peroxidase
PIN: PIN-formed
PYR: Pyrabactin resistance
QC: Quiescent center
QORL: Quinone oxidoreductase-like protein
qRT-PCR: Real-time quantitative polymerase chain reaction
RAM: Root apical meristem
RNA-seq: RNA-sequencing
RPKM: Reads per kb per million reads
RTCS: Rootless concerning crown and seminal roots
SAM: S-adenosylmethionine
SCR: Scarecrow
WRKY: WRKYGQK domain protein
