# Supplementary material for: Transcriptomic analysis reveals the gene expression profile that specifically responds to IBA during adventitious rooting in mung bean seedlings
Source: BMC Genomics. 2016 Jan 12;17:43. doi: 10.1186/s12864-016-2372-4 (PMC4709940; doi:10.1186/s12864-016-2372-4)
Supplement: Additional file 1: — Sequencing data information. (DOCX 13 kb) [file 12864_2016_2372_MOESM1_ESM.docx]

Additional file 1. Sequencing data information

|  | IBA6 | IBA24 |
| --- | --- | --- |
| Total Reads Count | 74549474 | 72937878 |
| Total Bases Count (bp) | 7454947400 | 7293787800 |
| Average Read Length (bp) | 100 | 100 |
| Q30 Bases Count (bp) | 5786082556 | 6523991267 |
| Q30 Bases Ratio (%) | 77.61 | 89.45 |
| Q20 Bases Count (bp) | 6482938876 | 6892957724 |
| Q20 Bases Ratio (%) | 86.96 | 94.50 |
| Q10 Bases Count (bp) | 6697291464 | 6981185220 |
| Q10 Bases Ratio (%) | 89.84 | 95.71 |
| N Bases Count (bp) | 10399403 | 17101522 |
| N Bases Ratio (%) | 0.139 | 0.234 |
| Good Bases | 6571259845 | 6930615090 |
| Good Sequences Count | 69082097 | 70805846 |
| Good Ratio (%) | 92.67 | 97.08 |
| Good Mean Length (bp) | 95.12 | 97.88 |
