# Supplementary material for: Transcriptomic analysis reveals the gene expression profile that specifically responds to IBA during adventitious rooting in mung bean seedlings
Source: BMC Genomics. 2016 Jan 12;17:43. doi: 10.1186/s12864-016-2372-4 (PMC4709940; doi:10.1186/s12864-016-2372-4)
Supplement: Additional file 9: — List of the genes and primers selected for q-PCR validation. (DOCX 22 kb) [file 12864_2016_2372_MOESM9_ESM.docx]

Additional file 9. Genes selected for q-PCR validation

| Gene ID | Functional description | Swissprot ID | Bitscore | E-value | RPKM | | |
| --- | --- | --- | --- | --- | --- | --- | --- |
|  |  |  |  |  | Con | Wat6 | Wat24 |
| Vr37254 | Auxin response factor 2, ARF2 | sp\|Q94JM3 | 952 | 0 | 89.852 | 37.954 | 50.760 |
| Vr35374 | Auxin response factor 18, ARF18 | sp\|Q653H7 | 816 | 0 | 26.952 | 16.698 | 14.999 |
| Vr35831 | Auxin response factor 1, ARF1 | sp\|Q8L7G0 | 885 | 0 | 29.128 | 15.349 | 16.768 |
| Vr29470 | Auxin response factor 3, ARF3 | sp\|O23661 | 450 | 1E-142 | 32.138 | 10.622 | 12.376 |
| Vr13756 | Auxin response factor 6, ARF6 | sp\|Q9ZTX8 | 376 | 2E-114 | 40.028 | 15.658 | 13.549 |
| Vr36957 | Auxin response factor 19, ARF19 | sp\|Q8RYC8 | 734 | 0 | 57.040 | 24.402 | 29.694 |
| Vr24483 | Auxin response factor 8, ARF8 | sp\|Q9FGV1 | 278 | 3E-82 | 13.053 | 8.386 | 8.696 |
| Vr23260 | Auxin transporter-like protein 4 | sp\|Q8L884 | 851 | 0 | 159.520 | 73.881 | 49.185 |
| Vr34322 | LOB domain-containing protein 41, LBD41 | sp\|Q9M886 | 185 | 3E-53 | 55.243 | 121.677 | 134.119 |
| Vr39013 | LOB domain-containing protein 21, LBD21 | sp\|Q9SRL8 | 188 | 1E-54 | 94.987 | 84.197 | 46.743 |
| Vr40540 | LOB domain-containing protein 29, LBD29 | sp\|Q9M2J7 | 186 | 3E-54 | 3.714 | 26.745 | 12.554 |
| Vr38129 | Sodium/hydrogen exchanger 2, NHX2 | sp\|Q56XP4 | 333 | 5E-103 | 381.908 | 429.401 | 472.443 |
| Vr40252 | NAC domain-containing protein 72, NAC72 | sp\|Q93VY3 | 350 | 2E-114 | 73.946 | 21.275 | 61.914 |
| Vr44893 | NAC domain-containing protein, NAC1 | sp\|Q84TE6 | 339 | 5E-112 | 24.715 | 17.499 | 19.606 |
| Vr56348 | NAC transcription factor, NAC2 | sp\|Q8GY42 | 303 | 3E-97 | 7.724 | 1.993 | 6.702 |
| Vr34452 | NAC transcription factor NAM-B2, NAMB2 | sp\|A0SPJ6 | 188 | 9E-53 | 105.389 | 35.786 | 100.297 |
| Vr32636 | Auxin-responsive protein IAA9, IAA9 | sp\|Q38827 | 404 | 8E-132 | 167.645 | 180.603 | 152.081 |
| Vr13386 | Auxin-responsive protein IAA26, IAA26 | sp\|Q8LAL2 | 227 | 9E-67 | 95.846 | 129.343 | 104.800 |
| Vr32189 | Auxin-responsive protein IAA14, IAA14 | sp\|Q38832 | 337 | 7E-111 | 630.691 | 551.345 | 220.222 |
| Vr13709 | Auxin-responsive protein IAA8, IAA8 | sp\|Q38826 | 207 | 1E-58 | 112.586 | 79.588 | 82.916 |
| Vr38087 | Alcohol dehydrogenase 1, ADH1 | sp\|P13603 | 618 | 0 | 7170.197 | 7091.752 | 3678.503 |
| Vr13399 | Alcohol dehydrogenase, ADH | sp\|P13603 | 307 | 5E-101 | 52.076 | 202.286 | 151.123 |
| Vr33063 | Histidine kinase 1, AHK1 | sp\|Q9SXL4 | 847 | 0 | 28.731 | 1.089 | 5.825 |
| Vr33358 | Histidine kinase 2, AHK2 | sp\|Q9C5U2 | 1275 | 0 | 20.795 | 12.318 | 11.801 |
| Vr40153 | Histidine kinase 3, AHK3 | sp\|Q9C5U1 | 1351 | 0 | 49.501 | 35.556 | 34.619 |
| Vr38180 | Dehydration-responsive protein RD22, RD22 | sp\|Q08298 | 310 | 2E-99 | 1249.984 | 356.157 | 738.560 |
| Vr34639 | Auxin-induced protein 22E, AUX22E | sp\|O24543 | 379 | 8E-129 | 52.651 | 50.996 | 26.642 |
| Vr31234 | Auxin-induced protein 22B, AUX22B | sp\|P32294 | 279 | 3E-88 | 58.082 | 58.968 | 37.863 |
| Vr39198 | Auxin-induced protein 22C, AUX22C | sp\|O24541 | 268 | 5E-89 | 6.366 | 43.768 | 4.778 |
| Vr47043 | Auxin-induced protein 15A, AUX15A | sp\|P33081 | 85.9 | 8E-20 | 1.261 | 39.716 | 2.638 |
| Vr22610 | Cationic peroxidase 1, PER1 | sp\|P22195 | 324 | 2E-107 | 0.193 | 139.508 | 372.845 |
| Vr21159 | Auxin efflux carrier component 1, PIN1 | sp\|Q9C6B8 | 464 | 2E-156 | 0.317 | 15.078 | 10.054 |
| Vr39799 | MYB transcription factor MYB134 | sp\|Q6R0H1 | 122 | 2E-28 | 249.382 | 5.232 | 112.908 |
| Vr40489 | MYB transcription factor MYB114 | sp\|Q6R0H1 | 118 | 5E-28 | 223.864 | 4.108 | 94.885 |
| Vr31128 | Cationic peroxidase 2 , PER2 | sp\|P22196 | 485 | 1E-168 | 4.195 | 169.179 | 226.783 |
| Vr36822 | Quinone oxidoreductase-like protein, QORL | sp\|Q9ZUC1 | 378 | 1E-124 | 7.085 | 40.927 | 4.776 |
| Vr39707 | Peptidyl-prolyl cis-trans isomerase CYP20, CYP20 | sp\|Q9ASS6 | 289 | 9E-94 | 58.173 | 62.356 | 58.411 |
| Vr38164 | Eukaryotic translation initiation factor 5A, eIF5A | sp\|Q9AXJ4 | 313 | 4E-105 | 606.853 | 602.711 | 602.154 |
| Vr31451 | Actin-related protein 4, ACTIN | sp\|Q84M92 | 712 | 0 | 18.727 | 19.322 | 20.725 |

Primers for q-PCR verification

Vr39707 F: 5' TCCCCAAACAGCCGAAAA 3' 59.5

Vr39707 R: 5' CCCCTTGAATCATGAAATCCTT 3' 59.9 107bp

Vr38164 F: 5' ACCTTAGGCTTCCAACCGAT 3' 58.0

Vr38164 R: 5' CTCCCATTGCAGACATAACAGA 3' 57.8 99bp

Vr31451 F: 5' CGTGTTTCCTTCTGTTGTTGG 3' 58.1

Vr31451 R: 5' CCTCTTTCCCTTAGCCTTGTC 3' 57.7 142bp

Vr37254 F: 5' ATCTCTCATCCGCAACACTCCT 3' 60.3

Vr37254 R: 5' TTCACCCCCACTTCTCTTACCTC 3' 61.4 123bp

Vr35374 F: 5' ACCTTGTCGCTGTCTTCGTGG 3' 62.4

Vr35374 R: 5' GTGGCGGCTGTTAAATTCCTC 3' 61.4 110bp

Vr35831 F: 5' CCGCAGCCAACACAAAGAAAC 3' 63

Vr35831 R: 5' GGTGATGGATAGAGGCCACGTC 3' 63.1 155bp

Vr29470 F: 5' CTTGTTCTAAGTGCCCTTGTGG 3' 59.3

Vr29470 R: 5' GCTTGCGTTTGCGTTTCCT 3' 61.3 175bp

Vr13756 F: 5' AAGGTTGGCTGGGATGAAT 3' 58.7

Vr13756 R: 5' TAAGGGGAAATGGAGATGGAT 3' 57.1 109bp

Vr36957 F: 5' GCTGGCTATTCTGCTGTACCTG 3' 59.5

Vr36957 R: 5' TCCCTTACTATTGGAGTCGGC 3' 58.7 96bp

Vr24483 F: 5' TTGAGGGAAAGTTGGAAGACC 3' 58.3

Vr24483 R: 5' CCTGAACCTAGAGCAAGGGATT 3' 59.5 196bp

Vr23260 F: 5' ACTCCAACGCCTTCTCTCTCCTC 3' 63.1

Vr23260 R: 5' ATCCCAATCACCTTCTCCCACA 3' 62.9 136bp

Vr34322 F: 5' CAATGGATAAGTTGCCCTCAC 3' 57.2

Vr34322 R: 5' ACCGCTGGTTGGAGATGG 3' 58.5 119bp

Vr39013 F: 5' CAAGGCTCAGAGACCCCG 3' 58.4

Vr39013 R: 5' TAGCAGCAGCGTGACATCG 3' 58.8 126bp

Vr40540 F: 5' ATCTCAGGGAACAGGCAGGTC 3' 60.4

Vr40540 R: 5' TCCGTCTGGAACCAACTCTGA 3' 60.2 121bp

Vr38129 F: 5' CTGAACTGCGAACCAATGC 3' 57.1

Vr38129 R: 5' TTCTTGCGTTTAGGAGGTGG 3' 58.0 136bp

Vr40252 F: 5' AGACCCTCTTGCCCAGTTG 3' 57.1

Vr40252 R: 5' CCGAAAATCGCCTTGCTT 3' 58.7 180bp

Vr44893 F: 5' AGGGTTCAGGTTCCATCCAA 3' 59.0

Vr44893 R: 5' GAATATCCCAAGGTTCGCACT 3' 58.5 125bp

Vr56348 F: 5' ATTCAACCTCAAAACCCCCT 3' 57.6

Vr56348 R: 5' CACAACACCCAATCATCAAGC 3' 58.5 76bp

Vr34452 F: 5' CCGACGAGGAATTGGTTGTG 3' 61.0

Vr34452 R: 5' GAGCGGTTGCCGTTAGGATA 3' 60.4 181bp

Vr32636 F: 5' GACTGTAGCGTCTGGTTCTTCG 3' 59.3

Vr32636 R: 5' CCTCAACTCCGTAGCCTTCAAA 3' 61.3 181bp

Vr13386 F: 5' ACCCCACCAAGAGAAAGGAAG 3' 59.8

Vr13386 R: 5' CCCACTAAGCGTGCAAATAAAA 3' 60.2 128bp

Vr32189 F: 5' GTGGAAACACCAAGGGCTACTG 3' 61.2

Vr32189 R: 5' CCAACCAACCACTTGAGCCT 3' 59.7 171bp

Vr13709 F: 5' TGGGCATAGGAGAGGAGGA 3' 57.5

Vr13709 R: 5' GAGAAAGACGGAACGGAGC 3' 57.0 163bp

Vr38087 F: 5' AGAATCCGCTGTTTCCTCGT 3' 58.8

Vr38087 R: 5' ACAGGTTGCTCTCTTCCGATT 3' 58.1 162bp

Vr13399 F: 5' AGGCTGGTGAAGTGCGTTT 3' 57.9

Vr13399 R: 5' TATGCGAGGAAACAATGGAGT 3' 57.4 101bp

Vr33063 F: 5' ACAAGAGCACCACCCCAAAA 3' 60.2

Vr33063 R: 5' TGTGTAAATCTTCGTGAAATGCC 3' 59.7 194bp

Vr33358 F: 5' GCATCAGGGGGGGTGTTTG 3' 62.9

Vr33358 R: 5' GCCTCAGCACGGCTTTTCAG 3' 63.2 125bp

Vr40153 F: 5' CAAATCGTCTGGGGGTTATCC 3' 60.8

Vr40153 R: 5' GAAGCAGTTTCTCCACCAATGAC 3' 60.5 144bp

Vr38180 F: 5' CCCGAGTCAGAGGAGGCTAA 3' 59.3

Vr38180 R: 5' CCCCGTTTCTTTGTCAACTTCT 3' 60.2 177bp

Vr34639 F: 5' AACTCTGATTCTTCGGACTCCACAAC 3' 64.3

Vr34639 R: 5' TCGCCCTGCTCCACCTTTTT 3' 64.3 143bp

Vr31234 F: 5' GCTCCCAGGAACAGAAGAAA 3' 56.8

Vr31234 R: 5' ATAAATCCCAGCACCCTCAT 3' 56.1 199bp

Vr39198 F: 5' TGGCCTCCGGTTTGCTCCTA 3' 65.1

Vr39198 R: 5' CCGCATCCTTCAACGCTTCG 3' 66.1 193bp

Vr47043 F: 5' TCAAGTTTTGGCAAGAAGCA 3' 57.0

Vr47043 R: 5' GTTCTCCCCAACATAGACAGC 3' 56.2 90bp

Vr22610 F: 5' CAAGAATCTCATCAACCAAAAGGG 3' 62.1

Vr22610 R: 5' GGGCTAAGGTTTCCCATTTTG 3' 60.5 156bp

Vr21159 F: 5' ATGCGGAGATTTACTCGCTTCA 3' 61.7

Vr21159 R: 5' CCCCCCCAAAACTACTCTGC 3' 61.0 132bp

Vr39799 F: 5' AAAAGGAAGAGCCCAACAAAG 3 '58.2

Vr39799 R: 5' CTGGAGAATAGCGCCTGAAA 3' 58.2 145bp

Vr40489 F: 5' GGACCTGCTTCATCTGTATCACTG 3' 60.5

Vr40489 R: 5' TGTTTGTGGTCTCCGTCATCTAA 3' 59.8 164bp

Vr31128 F: 5' CCTAGACACGGGTAGTCAAAGAGC 3' 61.6

Vr31128 R: 5' TCATCGTTCCAGAGGGCTTG 3' 60.7 102bp
